# Development and implementation of a highly-multiplexed SNP array for genetic mapping in maritime pine and comparative mapping with loblolly pine

**DOI:** 10.1186/1471-2164-12-368

**Published:** 2011-07-18

**Authors:** Emilie Chancerel, Camille Lepoittevin, Grégoire Le Provost, Yao-Cheng Lin, Juan Pablo Jaramillo-Correa, Andrew J Eckert, Jill L Wegrzyn, Diana Zelenika, Anne Boland, Jean-Marc Frigerio, Philippe Chaumeil, Pauline Garnier-Géré, Christophe Boury, Delphine Grivet, Santiago C González-Martínez, Pierre Rouzé, Yves Van de Peer, David B Neale, Maria T Cervera, Antoine Kremer, Christophe Plomion

**Affiliations:** 1 INRA, UMR1202 BIOGECO, F-33610 Cestas, France; 2 Université de Bordeaux, UMR1202 BIOGECO, 33170 Talence, France; 3 VIB, Department of Plant Systems Biology, Gent University, Technologie park 927, B-9052 Gent, Belgium; 4 Instituto de Ecología, Universidad Nacional Autónoma de México, Ciudad Universitaria, Circuito Exterior, Apartado Postal 70-275, México, D.F; 5 Department of Evolution and Ecology & Center for Population Biology, University of California at Davis, Davis, CA 95616, USA; 6 CEA, Institut de Génomique, Centre National de Génotypage, 2 Rue Gaston Crémieux, CP 5721, 91057 Evry Cedex, France; 7 INIA, Center of Forest Research (CIFOR), E-28040 Madrid, Spain

## Abstract

**Background:**

Single nucleotide polymorphisms (SNPs) are the most abundant source of genetic variation among individuals of a species. New genotyping technologies allow examining hundreds to thousands of SNPs in a single reaction for a wide range of applications such as genetic diversity analysis, linkage mapping, fine QTL mapping, association studies, marker-assisted or genome-wide selection. In this paper, we evaluated the potential of highly-multiplexed SNP genotyping for genetic mapping in maritime pine (*Pinus pinaster *Ait.), the main conifer used for commercial plantation in southwestern Europe.

**Results:**

We designed a custom GoldenGate assay for 1,536 SNPs detected through the resequencing of gene fragments (707 *in vitro *SNPs/Indels) and from Sanger-derived Expressed Sequenced Tags assembled into a unigene set (829 *in silico *SNPs/Indels). Offspring from three-generation outbred (G2) and inbred (F2) pedigrees were genotyped. The success rate of the assay was 63.6% and 74.8% for *in silico *and *in vitro *SNPs, respectively. A genotyping error rate of 0.4% was further estimated from segregating data of SNPs belonging to the same gene. Overall, 394 SNPs were available for mapping. A total of 287 SNPs were integrated with previously mapped markers in the G2 parental maps, while 179 SNPs were localized on the map generated from the analysis of the F2 progeny. Based on 98 markers segregating in both pedigrees, we were able to generate a consensus map comprising 357 SNPs from 292 different loci. Finally, the analysis of sequence homology between mapped markers and their orthologs in a *Pinus taeda *linkage map, made it possible to align the 12 linkage groups of both species.

**Conclusions:**

Our results show that the GoldenGate assay can be used successfully for high-throughput SNP genotyping in maritime pine, a conifer species that has a genome seven times the size of the human genome. This SNP-array will be extended thanks to recent sequencing effort using new generation sequencing technologies and will include SNPs from comparative orthologous sequences that were identified in the present study, providing a wider collection of anchor points for comparative genomics among the conifers.

## Background

Conifers represent over 670 species [1], some ranking as the largest, tallest, and longest living terrestrial organisms on Earth. They dominate many terrestrial landscapes, represent the largest terrestrial carbon sink, and provide significant ecological services to human societies. They are also of great economic importance, as they are primarily used for biomass, timber and pulp production worldwide. Domestication of conifers started about 60 years ago through traditional genetic improvement programs [2], which have resulted in substantial improvements of overall growth, wood quality, pest resistance and adaptation to present and future climatic conditions. However, the long generation intervals and the difficulty of accurately evaluating traits in early stages have hampered progress through breeding [3]. Furthermore, their extraordinary large genome size (seven times higher than the human genome, [4]) prohibits classical map-based cloning approaches of major-effect quantitative trait loci (QTL).

During the past 20 years, many sophisticated genomic tools have been developed to accelerate the domestication process by exploring in depth their genetic diversity. Linkage maps represent a major tool in genomic-based breeding because of their central role in dissecting the genetic architecture of quantitative trait variation [5]. Moreover, the relatively well conserved genome organization in conifers makes it possible to transfer genetic information from one species to another by comparative mapping (e.g. [6]). Genetic mapping activities, therefore, will continue to dominate research in conifer genetics [7] at least until a conifer genome sequence has been completely decoded.

The progress of genetic mapping in conifers is reflected by the development of different generations of molecular markers. The first (RFLP: Restriction Fragment Length Polymorphism) and second generation (RAPD: Random Amplified Polymorphic DNA and AFLP: Amplified Fragment Length Polymorphisms) markers made it possible to construct genetic linkage maps for twelve pine (*Pinus taeda, P. pinaster, P. sylvestris, P. strobus, P. radiata, P. palustris, P. elliottii, P. edulis, P. densiflora, P. contorta, P. caribaea*, and *P. brutia*) and four spruce species (*Picea abies, P. glauca, P. mariana *and *P. rubens*) (reviewed by [7]). Furthermore, over the last decade, the increasing availability of expressed sequence tags (ESTs) has provided a valuable source of new PCR-based molecular markers, such as EST-derived microsatellites (EST-SSRs), EST-based polymorphisms (EST-Ps) and single nucleotide polymorphisms (SNPs). Because of their abundance, higher availability and stability compared with simple sequence repeats (SSRs), and the ease with which data can be standardized due to their bi-allelic nature, SNP markers provide enhanced possibilities for genetic and breeding applications, such as construction of linkage maps and detection of genotype/phenotype associations [8]. Several SNP genotyping approaches have been developed in the last few years. These approaches vary in terms of sensitivity, reproducibility, accuracy, capability of multiplexing and throughput [9,10]. Among the SNP genotyping technologies available to date, the GoldenGate assay developed by Illumina has demonstrated high success rates within plant genomes of differing complexities, such as pea [11], soybean [12], barley [13], maize [14], spruces [15] and pines [16-18].

In the present study, a custom GoldenGate assay containing 1,536 SNPs derived from gene sequences was designed to genotype two maritime pine (*Pinus pinaster *Ait., 2n = 2x = 24) mapping populations, while earlier studies have heavily relied on anonymous markers such as RAPDs [19] and AFLPs [20-22]. The information contained within each individual map was then synthesized into a single consensus map, from which a set of orthologous markers allowed the alignment with the reference *Pinus taeda *linkage map. The present study is therefore an important step for advancing comparative genomics in conifers.

## Methods

### Mapping populations

Two maritime pine mapping populations were used in this study: (1) a three-generation outbred pedigree (G2) (parental accessions 9.106.3 and 10.159.3; [6,21]), comprising 201 offspring, and (2) a three-generation inbred pedigree (F2) obtained by the self pollination of an inter- "Landes × Corsica" provenance hybrid (accession H12 resulting from the control cross between L146 and C10 genotypes), resulting in a total number of 500 offspring. Trees of both mapping populations were measured for a series of quantitative traits related to biomass production and wood properties, for which some QTLs have already been detected ([23] for the G2 and in progress for the F2 population). The sample size used for mapping was 89 and 88 offspring for the G2 and the F2 pedigree, respectively.

### DNA extraction and quality control

Young needles were harvested in spring and conserved at -20°C before DNA extraction. Pieces of frozen needles (around 30 - 40 mg) were crushed using a mixer mill (Retsch MM300, Haan, Germany). Isolation of DNA was performed using *Invisorb DNA plants 96 kit *from Invitek (GmbH, Berlin, Germany) following kit's specific instructions. All concentrations were measured using the fluorescence assay (Quant-IT kit, Invitrogen, Carlsbad, CA, USA). Low values were systematically checked by a second measurement, and all samples with high initial concentrations were diluted to concentrations ranging from 150 to 170 ng/μL. In addition, DNA quality was checked on a subset of samples (about 10%) by loading DNA on agarose gel to estimate its integrity. All samples that did not met the concentration and quality criteria recommended by Illumina for GoldenGate assay were excluded from further steps.

### EST production and assembly

A total of 40,774 ESTs were obtained from wood forming tissue, root, needles and vegetative bud (Additional file 1). Base calling and quality assessment were determined using the Phred software [24]. Vector and adaptor sequences were trimmed using cross-match [25] and custom Perl scripts. As sequence data vary in quality, an automatic inspection was performed and sequences > 60 bp long and containing more than 90% of nucleotides with high quality (Phred score > 20) were kept. Finally, 31,678 informative reads were used for the assembly using stackPACK™ [26,27], resulting into a unigene set comprising 4,483 contigs (including 22,431 ESTs) and 9,247 singletons.

### SNP discovery

A first set (set 1, Table 1) of *in vitro *polymorphisms was detected in 49 different gene fragments involved in plant cell wall formation or drought stress resistance (Additional file 2). For each fragment, an average of 50 megagametophytes (haploid tissue surrounding the embryo) from different populations covering the natural range of the species were sequenced (see additional file 3 for the exact detection depth, and additional file 2 for the detail of populations that were sequenced for each fragment). The chromatograms were aligned with CodonCode Aligner (Codon Code Corporation, Dedham, MA) and visually checked. Nucleotides with Phred scores < 30 were considered as missing data. The use of megagametophytes lowered the risk of confusing polymorphism at a unique locus with differences between paralogous loci, as amplification of two or even more members of a gene family would have been easily detected by the visualization of double peaks in the "haploid" chromatograms. A second set (set 2, Table 1) of *in vitro *polymorphisms was detected in 392 different amplicons re-sequenced in the frame of the CRSP project [28]. The discovery panel in this case consisted of 12 megagametophytes (also covering the natural range of the species), including the parents of the G2 and F2 progenies. Sanger sequencing was externalized (Agencourt Biosciences, Beverly, MA, USA) and followed custom protocols in 384-well format. The SeqQual pipeline [29] was used to assemble the sequences for each fragment and to mask nucleotides with Phred scores below 30. The chromatograms and alignments were visually checked and only those resulting in a single, well-aligned contig without double peaks at polymorphic sites were kept for further applications. Then, we used the Perl script *snp2illumina *[18], to automatically extract polymorphisms (bi-allelic SNPs and bi-allelic Indels from one to six base pairs) and output them as a SequenceList file compatible with the Illumina Assay Design Tool (ADT) software [30]. The *snp2illumina *script also recorded for each polymorphism the number of sequences considered for the detection (detection depth) and the minor allele frequency (MAF), two parameters that proved critical for high validation rate of EST-derived SNPs [18,31]. The minimum detection depth was set to four. Singletons were discarded in order to minimize the number of false positives, as it is highly unlikely to obtain sequencing errors twice at the same base location.

**Table 1:** Number of polymorphisms detected and selected in the different sequencing sets.

		Number of polymorphisms (number of fragments or contigs represented)		
		
	Number of fragments or contigs available for detection	Detected	Functionality score ≥ 0.4	Selected for the assay
Validated SNPs^a^	-	-	-	229 (132)
*in vitro *polymorphisms, set 1	49	572 (49)	352 (48)	37 (19)
*in vitro *polymorphisms, set 2	392	1,678 (375)	1,015 (367)	560 (325)
*in silico *polymorphisms, set 3	1,532	4,376 (587)	1,431 (518)	710 (424)

TOTAL	1,967	6,626 (1005)	2,798 (927)	1,536 (885)

^a ^From Lepoittevin *et al*. (2010) including 110 SNPs detected in 35 fragments from *in vitro *polymorphisms, set 1.

Finally, a set of *in silico *polymorphisms (set 3 in Table 1) was detected in the maritime pine EST assembly. Only the 1,532 unigene elements containing at least four sequences were considered. The SeqQual pipeline was also used to mask nucleotides with Phred scores below 30, and the *snp2illumina *script was used for automatic polymorphism detection (with the same criteria as cited above, i.e. detection depth ≥ 4 and no singleton).

### SNP selection for customized 1,536-GoldenGate array construction

For the 1,536-SNPs GoldenGate array design, we first included 229 SNPs previously validated on maritime pine Aquitaine populations with the same genotyping technology [18], including 110 *in vitro *polymorphisms from 35 of the 49 re-sequenced candidate gene fragments previously described, and 119 *in silico *SNPs detected in a maritime pine unigene assembly. For the additional 1,307 SNPs, priority was given to *in vitro *polymorphisms. We only included polymorphisms showing designability score above 0.4, which is the lower limit for genotyping success according to the manufacturer. This score is provided by ADT software and is similar to a predicted probability of genotyping success, taking into account the sequence conformation around the SNP and the lack of repetitive elements in the surrounding sequence [32]. Following Illumina's recommendation, the main technical constraint was that the selected polymorphisms should be separated by at least 60 nucleotides from each other. When several polymorphisms occurred within this limit, it was decided to filter out the lowest frequency variants and polymorphisms showing high level of linkage disequilibrium with other selected polymorphisms of the same fragment. Finally, with the goal of mapping as many different loci as possible, emphasis was given to breath of coverage (i.e. 1 SNP per gene for a large number of genes) *versus *depth of coverage (i.e. as many SNPs as possible per gene for a low number of genes). We used BlastN analysis [33] to ensure that *in vitro *and *in silico *polymorphisms belonged to different genes. We also blasted each amplicon to avoid including in the array members of the same gene family that had high homology in the sequence surrounding the target SNP.

### SNP genotyping assay

**Figure 1:**
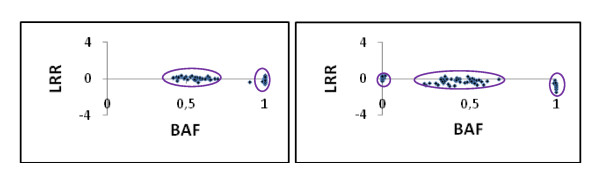
**Scatter plots representing the Log R ratio (LRR- y axis) and the B allele frequency (BAF- x axis)**. On the left panel, classical pattern with two clusters for a SNP segregating in the 1/2: 1/2 ratio (one homozygous class and one heterozygous class). On the right panel, classical pattern with three clusters for a SNP segregating in the 1/4: 1/2: 1/4 ratio (two homozygous classes and one heterozygous class).

Genotyping of SNPs was carried out at the French National Genotyping Center (CNG, Evry, France) using the Illumina GoldenGate assay according to the manufacturer's instructions (Illumina Inc., SanDiego, CA, USA). This assay is based on the use of 2 allele-specific and one locus-specific oligonucleotide per SNP locus. After the step of allele specific extension and ligation, a PCR reaction is performed with universal primers labeled with either Cy3 or Cy5 dye to distinguish each SNP allele. Fluorescent products are hybridized to precoded beads on an array matrix. The fluorescent signals are read using the Illumina Bead Array Reader software. From the initial set of 1,536 SNPs, 480 (31.25%) were excluded by the CNG quality control procedure, because of weak or ambiguous signal. The remaining 1,056 SNPs (68.75%) were individually inspected after visualization of the B allele frequency (normalized measure of the allelic intensity ratio of the two alleles) versus the Log R Ratio (normalized measure of the total signal intensity for two alleles of the SNP) to screen out non-segregating markers in the studied pedigrees. For each SNP, a homozygous genotype displays a signal in either the Cy3 or Cy5 channels, and a heterozygous genotype displays signals in both channels. The SNPs for which 2 or 3 scatter plots (depending on the type of segregation) were clearly identified, were classified as "polymorphic SNPs" (Figure 1). Then, a total of 89 and 88 offspring were genotyped for the G2 and F2 mapping populations, respectively.

### Linkage mapping strategy

#### G2 map construction

Linkage analysis was conducted using the "two-way pseudo-testcross" mapping strategy [34] and independent linkage maps were constructed for each parental tree. Polymorphic SNP markers were classified into two categories according to their segregation proportions: (1) test-cross markers segregating 1/2: 1/2 (heterozygous in one parent and homozygous in the other) or (2) inter-cross markers segregating 1/4: 1/2: 1/4 (heterozygous in both parents). The polymorphic SNPs were combined with 619 other markers (including AFLPs, SSRs, EST-Ps and additional SNPs; see additional file 4) available from previous studies [6,21,23]. Mendelian segregation ratios were tested using χ^2^-square tests (P < 0.01). Linkage analyses and map estimations were performed with JoinMap v 4.0 [35] using CP (Cross Pollinators) as population type. Recombination frequencies were converted into map distances in centiMorgans using the Kosambi mapping function [36]. All available markers were first grouped at a minimum LOD threshold of 3.0. Linkage groups (LGs) were named according to the nomenclature used for loblolly pine, as homologous LGs to this reference species were identified (see Results section). Once the different groups were determined, the female and male maps were built independently using the regression algorithm. The procedure is basically a process of building a map by adding loci one by one, starting from the most informative pair [35]. When using a high number of markers genotyped on a variable number of offspring, this procedure generally leads to the construction of 3 different maps with decreasing statistical support (denoted as map1, map2 and map3), indicating difficulties to reliably order markers within linkage groups. Because the different types of markers were genotyped on different number of samples (ranging from 46 to 201 trees; see additional file 4), we followed a framework mapping approach, where framework markers segregating 1/2: 1/2 (ordered with the highest statistical support, map 1) were selected (mainly among AFLPs genotyped on 201 F1s) and used as anchor points to localize poorer fitting loci (accessory markers). The relative position of each accessory marker to its most probable framework marker was then provided using the two-point LOD scores and recombination fractions available in the "maximum linkage" table of JoinMap.

Total genome size, G, was estimated from Hulbert *et al*. [37] modified by Chakravarti *et al*. ([38]; Method 3), as G = [N*(N-1)*X]/K in which N represents the number of framework markers in the map, X is the maximum observed map distance between two markers for which the expected value of LOD score is z (here 3, 4 and 5) and K is the observed number of locus pairs with LOD score ≥ z.

#### F2 map construction

**Figure 2:**
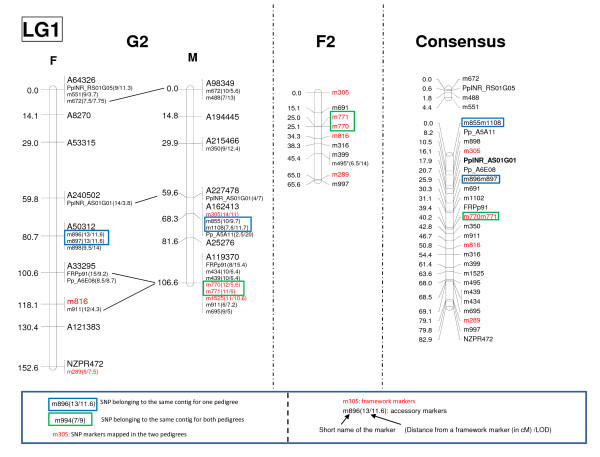
**Example of linkage group (LG1) for the Female and Male G2 maps and its counterpart in the F2 and consensus maps in maritime pine**.

For the F2 pedigree, no previous marker data were available, therefore the map only relied on the SNPs segregating 1/4: 1/2: 1/4 (heterozygous in the F1 parent). Linkage analysis was performed with JoinMap v 4.0 using F2 as population type and the regression algorithm. In many cases, all the markers belonging to a given LG were ordered with a minimum LOD score of 3.0, except for linkage groups 1, 2, 7 and 9 for which some markers had to be excluded (and therefore placed as accessory markers) if the threshold LOD score of 3.0 was to be maintained (Figure 2 for LG1 and additional file 5 for all the LGs). Genome size was estimated as explained above, with N representing the number of unique framework SNP markers.

### Consensus map construction

One of the objectives of this study was to construct a consensus map of the species based on SNP markers. To this end, the two datasets (except AFLP markers for the G2 pedigree) were combined assuming homogeneous recombination rates between the two studied populations and between the male and female linkage maps of the G2 mapping population (but see [39]). The construction of an integrated map required the estimation of a single recombination rate for each SNP marker pair based on all available meioses, irrespective from which crosses the genotypic data had been derived. Heterogeneity of recombination rate between SNP marker pairs was tested between the two pedigrees using the "Heterogeneity test" implemented in JoinMap v 4.0.

### Comparative mapping between maritime and loblolly pines

Homology between loblolly (*Pinus taeda*) and maritime pine sequences, from which SNPs were derived for linkage mapping in maritime pine, was established using sequence similarity as assessed with MegaBlast [40]. Resequenced data from maritime pine were queried against the 5,811 EST contigs in loblolly pine, of which approximately 20% are located on its consensus linkage map. Visual inspection of Blast results was carried out to eliminate results due to small regions of homology (i.e. hit coverage in *P. pinaster *< 75%), as was observed among different members of the same gene family. In this case, a small to moderate portion (< 75% of the sequence length) of the maritime pine sequence was similar to multiple loblolly pine EST contigs. These EST contigs in loblolly pine also shared strong sequence homology (e.g. 90% identity) among themselves in the same region as the Blast hit, suggesting that this region was conserved across paralogs. In these cases, the loci were excluded because orthology was difficult to establish.

This *a posteriori *approach yielded poor results (see Results section). With the main goal of providing *a priori *a wider set of comparative anchor sequences (COS) between the two most studied gymnosperm genera (*Pinus *and *Picea*) we carried out the following bioinformatic analysis. The pine and spruce unigene datasets were downloaded from the TGI (The Gene Index project) version 7.0 and 3.0 with 61,864 and 80,494 unique sequences, respectively. Single copy COS markers were detected using a two-step approach, starting with the filtering of paralogous sequences within genera, followed by the identification of conserved sequences between genera. In the first step, single copy genes within species were identified as follows: (1) each unigene was self-BlastN against itself using parameters of word size 28, penalty for a nucleotide mismatch -2, reward for a nucleotide match 1, filter off and the e_value < 1e-15 with MegaBlast search, (2) we then used the TribeMCL clustering algorithm [41] to identify homolog sequences in each dataset. Based on the TribeMCL clustering result, we defined the sequence that did not form a gene family with other sequence(s) as a singleton. In the second step, shared single-copy genes between *Pinus *and *Picea *were searched by joining singletons from both genera into one dataset and self-BlastN using the same parameters as described above. Alignments for which coverage was less than 60% of the shortest sequence and identity did not exceed 80% were removed. TribeMCL clustering was then conducted on the post-processed BlastN result to construct gene families. A COS marker was finally defined as a sequence presenting one single unigene from *Pinus *and *Picea *within the same family.

The COS marker dataset was finally searched against the maritime pine EST assembly to identify the orthologous sequences in our dataset. In order to eliminate false-positive COS markers, the pine and spruce unigenes were searched against two plant protein databases using BlastX: PLAZA ([42], containing *Populus trichocarpa, Arabidopsis thaliana, Carica papaya, Vitis vinifera, Oryza sativa, Sorghum bicolor, Physcomitrella patens, Chlamydomonas reinhardtii and Ostreococcus lucimarinus *protein-coding genes; [43]) and NCBI non-redundant (nr) protein database. COS markers were divided into five categories according to the presence or absence of hits in the queried databases, providing a range of confidence level in representing the COS markers: COS markers in category A display best hits either with PLAZA or NCBI nr protein databases; in category B, COS only hit in the NCBI nr protein database; for category C, COS are only found in the maritime pine unigene set; for category D, COS are only present in the pine and spruce TGI unigene; and in category E, COS most likely correspond to paralogs or to alternative splice events in the pine and spruce genome. Thus, these five categories provide a range of sequence conservation and possible duplication history of COS markers (from A - conserved with other plant genomes to D - conifer specific and E - duplicates).

## Results

### SNP array construction

A total of 2,250 *in vitro *SNPs (572 from candidate genes and 1,678 from the CRSP resequencing project) and 4,376 *in silico *SNPs were detected in the different data sets (Table 1). About 61% of the *in vitro *SNPs had acceptable designability scores (≥0.4), as opposed to 33% for *in silico *SNPs. A 1,536 GoldenGate SNP array was built from the 229 SNPs (including 110 *in vitro *and 119 *in silico *polymorphisms) located in 132 different gene fragments previously validated in a GoldenGate array on the maritime pine Aquitaine populations [18], the 597 *in vitro *SNPs detected in either resequenced candidate genes (37 SNPs in 19 genes) or in data from the CRSP resequencing project (560 SNPs in 325 amplicons), and, the 710 *in silico *SNPs detected in 424 contigs from the maritime pine EST assembly. These 1,536 SNPs were distributed in 885 non-redundant gene fragments or contigs.

### SNP genotyping statistics

Out of the 1,536 SNPs, 707 and 829 markers were classified as *in vitro *or *in silico *SNPs (see Methods section). The average sample success rate (i.e. the percentage of successfully genotyped SNPs per DNA sample) was 86.5% and 88.4% for the G2 and the F2 mapping populations, respectively. After the first screening step which excluded failed markers, a total of 529 (74.8%) *in vitro *and 527 (63.6%) *in silico *SNPs remained and were made available through the NCBI database ([44], see additional file 3 for accession numbers). There was a statistical association between SNP origin (*in vitro *vs. *in silico*) and the proportion of failed SNPs, which was greater for *in silico *SNPs [χ^2 ^= 21.97, *P *= 2.77 × 10^-6^].

Before the construction of the SNP bead array, a designability score (called SNPScore) was given to each SNP by the Illumina Assay Design Tool. This score ranges from 0 to 1, with higher values reflecting greater ability to design a successful assay, and 0.6 being the lowest threshold recommended by Illumina to design the GoldenGate OPA (Oligo Pool Assay). In our study, the mean Illumina SNPScore for successfully genotyped SNPs (i.e polymorphic and monomorphic SNPs) and failed SNPs were 0.85 [min: 0.4; max: 1] and 0.75 [min: 0.4; max: 1], respectively, which indicates that 94.1% of the successful and 78.3% of the failed SNPs corresponded to Illumina's recommendation (i.e SNPScore ≥ 0.6). A Chi-square test for independence confirmed earlier findings [18] that there is a significant correlation between SNPScore and the success rate for the conversion of a SNP into a successful genotyping assay [χ^2 ^= 83.78, *P *= 2.2 × 10^-16^]. We also found a higher proportion of SNPs with SNPScore ≥ 0.6 for *in vitro *than for *in silico *SNPs [χ^2 ^= 22.71, *P *= 1.88 × 10^-6^], suggesting that failed assays for *in silico *SNPs were due, at least in part, to lower designability scores. This being said, it should be pointed out that several SNPs with relatively low SNPScore (0.4-0.6) were still successful. A relaxed criterion could thus be attempted especially for those non model species for which few SNPs are available or for some SNPs in candidate genes of interest.

### Identification of informative SNPs

**Figure 3:**
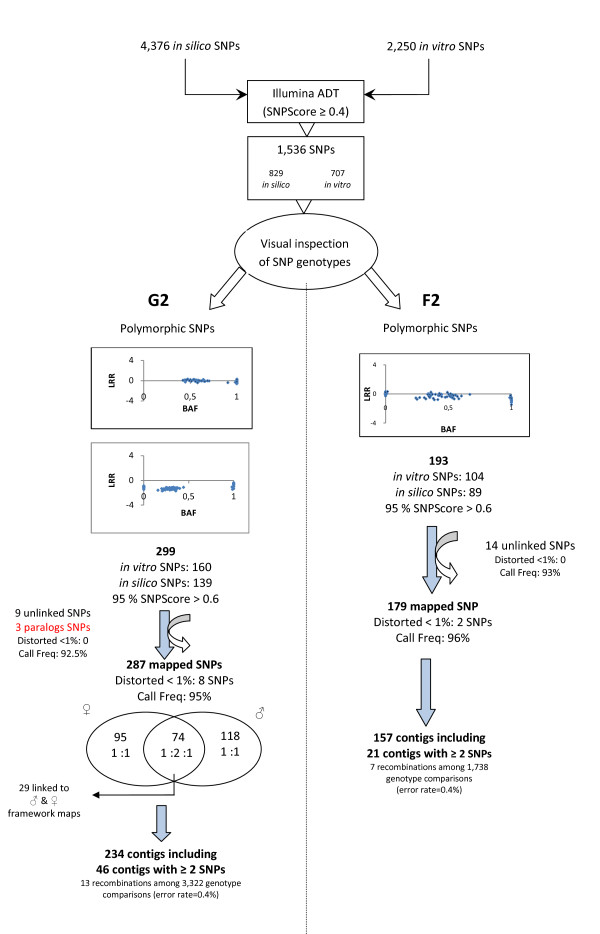
**Schematic representation of the mapping strategy for the G2 and F2 pedigrees in maritime pine**. BAF means B allele frequency (normalized measure of the allelic intensity ratio of the two alleles) and LRR means the Log R Ratio (normalized measure of the total signal intensity for two alleles of the SNP).

After visual inspection of successfully genotyped SNPs that allowed assignment of individual trees to their respective genotypic classes, we declared 299 and 193 polymorphic SNPs for the G2 and F2 mapping populations, respectively (Figure 3). The conversion rate, corresponding to the number of polymorphic SNPs divided by the total number of SNPs in the assay [45], was 19.5% and 12.5%, for the G2 and F2 populations, respectively. A total of 394 SNPs were available for mapping. While 296 were pedigree specific, 98 SNPs were shared between both populations. In the G2 mapping population, there were 160 polymorphic *in vitro *SNPs (30.3% of the successfully genotyped SNPs) and 139 polymorphic *in silico *SNPs (26.4%). In the F2 mapping population, 104 of the polymorphic SNPs came from the *in vitro *set (19.7%) and 89 from the *in silico *set (16.9%). There was no correlation between the SNP origin and the proportion of polymorphic SNPs [χ^2 ^= 1.7, *P *= 0.18 for the G2 and χ^2 ^= 1.18, *P *= 0.28 for the F2 pedigrees].

### Validation of the SNP genotyping assay

The presence of several SNPs within the same amplicon (for *in vitro *SNPs) or contig (for *in silico *SNPs) provided a unique opportunity to validate the genotyping assay by checking the inconsistency between offspring genotype assignments of physically linked SNPs. For the G2 population, 40 contigs presented more than one SNP. From a total of 3,322 data points we only detected 13 recombination events between SNPs belonging to the same contig, which translates to a genotyping error of 0.4%, if we consider that the probability of crossover within a contig of a few hundred base pairs long is null. For the F2 population, there were 21 contigs containing more than one SNP totalizing 1,543 data points, from which 7 recombination events were observed (i.e. 0.4% error rate). These results confirm the GoldenGate assay reproducibility in maritime pine as previously reported [18].

### Construction of individual genetic linkage maps

For the G2 mapping population, 287 (from which 8 were distorted, *P *< 0.01) out of 299 polymorphic SNPs were mapped with a LOD ≥ 3.0 on the female and male parental maps (Figure 2, additional file 5). This map includes 213 markers segregating 1:1 (95 mapped on the 13 LGs of the female map and 118 on the 14 LGs of the male map) and 74 markers segregating in the 1:2:1 ratio. From these 74 shared markers, 29 were mapped on both parental maps. Overall, these 287 SNPs represented 234 different contigs. For the F2 pedigree, 179 out of 193 polymorphic SNPs were mapped onto 21 LGs that aligned subsequently to the G2 LGs by using the 98 common markers between both pedigrees as anchor points. These 179 SNPs (2 were distorted, *P *< 0.01) represented 157 different contigs. The main features of the G2 (female and male) and F2 maps are summarized in Table 2.

**Table 2:** Statistics of individual and consensus linkage maps for the G2 and F2 mapping populations.

	G2	F2	Consensus
Number of linkage groups	13 (♀), 14 (♂)	21	24
Type of markers used for mapping	AFLP, EST-P, SSR, SNP	SNP	EST-P, SSR, SNP
Number of polymorphic SNPs	299	193	
Number of mapped SNPs	287	179	357
Number of mapped contigs	234	157	292
Number of unlinked SNPs	12	14	10
Number of distorted mapped SNPs (P < 1%)	8	2	

### Comparative mapping between F2 and G2 linkage maps

A total of 98 SNPs (belonging to 89 contigs) were mapped in both mapping populations. Their distribution along the 12 LGs is shown in additional file 6, and varies from 1 anchor marker for LG8 to 12 for LG2. On average, homologous LGs between the F2 and G2 maps were assigned based on 8 common SNPs (from 7 common contigs) per LG. Marker order between the two maps was generally consistent when taking into account the regions covered by homologous markers (Figure 2, additional file 5). Only six marker pairs showed significant heterogeneity in recombination rate at the 1% level, out of the 457 pairwise comparisons derived from SNPs simultaneously mapped in both populations, which is close to the number expected by chance alone. This result suggests that recombination fraction is homogeneous between the two studied populations, and that the consensus map is a reasonable approximation of the marker order that can be used for comparative mapping.

### Consensus map construction

When the three individual maps were merged into a single consensus map, 357 SNPs (from 292 contigs) were mapped onto 24 LGs, with a minimum of 18 SNPs in LG8 and a maximum of 42 SNPs in LG5. The 24 consensus LGs covered a distance of 1,320 cM, with a length ranging from 78 to 145 cM, and the distance between adjacent markers (comprising all types of markers without accessory markers) averaging 4 cM. The marker order was generally well conserved between the individual and consensus maps.

### Genome size estimation

Observed map lengths ranged from 869 cM for the F2, to 1,571 and 1,726 cM for the G2 male and female maps, respectively. Estimated genome sizes computed for different statistical support for linkage (LOD 3-4-5) varied from 2,490 to 2,700 cM for the G2 female map, from 2,400 to 2,765 cM for the G2 male map, and from 2,238 to 3,252 cM for the F2 map, with an average of 2,500 cM. Given a physical size of 51.5 pg/2C [21], 1 cM would correspond to 10.3 Mb/cM on average.

### Comparative mapping between maritime and loblolly pines

**Figure 4:**
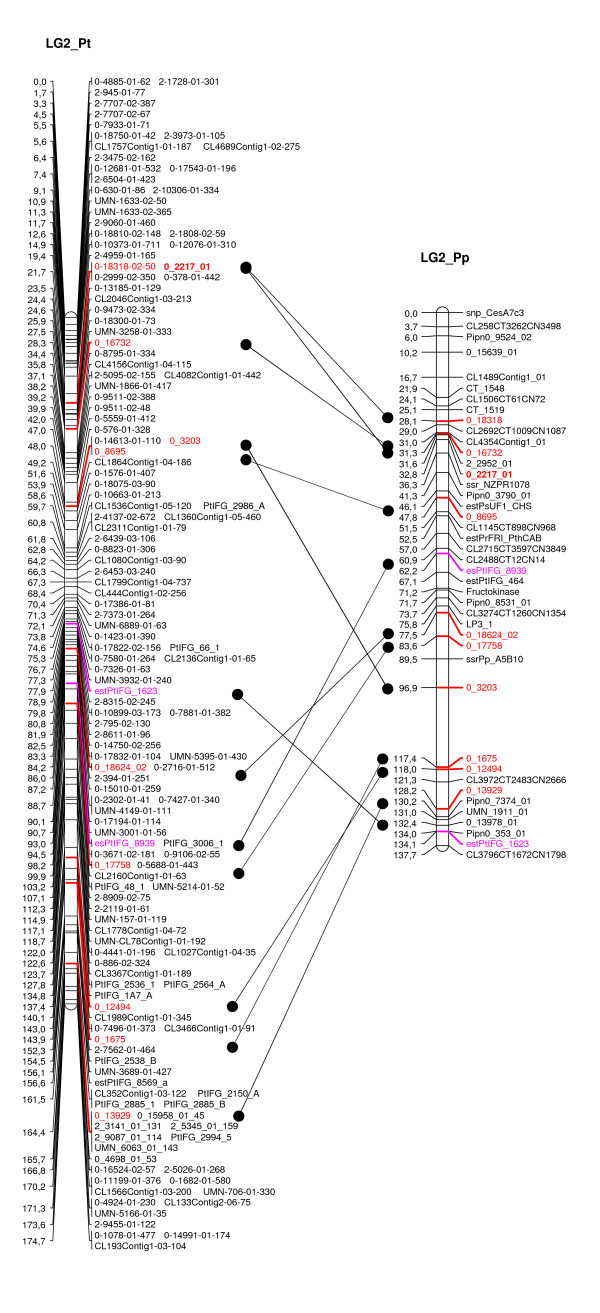
**Example of homologous linkage group between loblolly (LG2_Pt) and maritime (LG2_Pp) pines**. Orthologous SNP markers are in red and EST-P markers in pink.

Based on a stringent sequence homology search between the 292 mapped contigs in maritime pine (this study) and the 1,489 mapped contigs in loblolly pine (published as supplemental data in [46]), 50 COS markers were identified, from which 46 made it possible to align 7 linkage groups (additional file 7) between both species maps with at least 3 orthologous markers. In four cases, there were inconsistencies in the linkage group assignments of SNP markers, which could be attributed to paralogy rather than to alternative splice events, as they were found in different linkage groups. Taking into account the EST-P markers that were already identified as anchor points between these two species [6], all linkage groups are now aligned. Figure 4 shows an example of homologous linkage groups between loblolly and maritime pines anchored by 12 COS markers (10 SNPs, 2 EST-Ps). Overall, the total number of anchor loci amounts to 77 markers.

### Identification of COS markers between Pinus and Picea

A total of 22,434 and 19,606 single-copy genes from pine and spruce TGI unigenes were identified in the first clustering attempt. A second attempt, with more stringent BlastN filtering parameters, resulted into 1,265 COS markers (additional file 8), that were split into five categories: (1) 1,087 COS markers presenting a best hit either with plant or NCBI nr protein database, (2) 16 COS markers showing a hit only with the NCBI nr database, (3) 22 COS markers with no hit with both protein databases but showing at least one homolog with the maritime pine dataset, (4) 74 COS markers only present in the pine or spruce TGI unigenes, and (5) 66 COS markers potentially corresponding to duplicated genes within the pine or spruce genomes. Markers in the first class should be considered as the main target for further development in view of comparative genomic surveys in conifers.

## Discussion

### Genotyping assay

The global success rate (i.e percentage of SNPs successfully genotyped, considering both monomorphic and polymorphic SNPs) obtained for the SNP array developed in the present study was 68.75%. This result is similar to that achieved for a 384-GoldenGate SNP array on the same species (66.9%; [18]). Such similar success rate indicates that higher multiplex levels of GoldenGate arrays do not affect the assay's effectiveness. However, the success rate was statistically different between in *vitro *(74.8%) and *in silico *(63.6%) SNPs. There are many causes of genotyping failures in EST-derived SNPs [31]. First, in EST sequences, sequencing errors can lead to the identification of false-positives. In the present study this factor was however minimized by setting a minimum detection depth of 4 sequences for *in silico *SNPs and by removing singletons. Second, low quality of SNP flanking sequences, non-identified polymorphisms nearby the targeted SNP and the possible presence of exon-intron junctions close to targeted polymorphic sites can also lead to genotyping failures. Screening for Phred scores above 30 allowed coping with the former source of failure, while the two latter were controlled for *in vitro *polymorphisms only and may have contributed to the observed difference in success rate. Finally, as indicated by Shirasawa *et al*. [47], failed SNPs may be designed on multi copy genes and may disrupt the fluorescent signal cluster on the analysis software. Because *in vitro *SNPs were obtained from single copy locus (see Methods), *in silico *SNPs were certainly affected to a greater extent by this problem.

The conversion rate (i.e percentage of polymorphic SNP) in this study was 19.5% for the G2 and 12.5% for the F2 mapping populations. We did not find statistical differences in terms of percentage of polymorphic SNPs between *in vitro *and *in silico *derived SNPs. In fact, SNPs were not *a priori *screened for their polymorphism in the mapping pedigrees, which resulted in a relatively low percentage of polymorphic markers. These figures are quite similar to those obtained by Jermstad *et al*. [17] for two sugar pine mapping populations, which had a conversion rate of 12.3% and 18.4%. On the other hand, higher SNP conversion rates (69.2% and 77.1%) were obtained by resequencing amplicons of parental lines of mapping populations of white and black spruce, respectively [15]. Such an approach is thus an efficient method for tracking informative SNPs for linkage analyses [17,48].

### Consensus linkage map and genome size (in cM) estimation

The genome size of *Pinus pinaster *was first estimated from low coverage protein based linkage maps by Gerber and Rodolphe [49] who obtained sizes ranging from 1,400 to 2,300 cM depending on the method and the data set used. Later on, Echt and Nelson [50] narrowed down this estimate to 1,880-2,084 cM with an extended marker dataset. In the present study, genome size was estimated for two other mapping populations and three different LOD score thresholds following method 3 of Chakravarti *et al*. [38]. On average, our genome size estimate amounted 2,500 cM, which is higher than earlier reports on the same species but in the range of estimates obtained for other pines from partial linkage data: 2,639 cM for *Pinus sylvestris *[51], 2,612 to 2,656 cM for *Pinus palustris *[52] and 2,407 cM for *Pinus contorta *subsp. latifolia [53].

Comparing genome size estimates between three species, *Pinus pinaster*, *Pinus strobus *and *Pinus palustris*, Echt and Nelson [50] showed that pines have similar genome lengths, suggesting a highly conserved genomic rate of recombination across species. The differences in genome size estimations observed in the literature may be due to the choice of the mapping function and to variations in the recombination rates of pollen and seed parents [39,54]. Furthermore, the use of different methods to estimate genome size, such as method 2 of Hulbert *et al*. [37,38] or method 3 modified by Chakravarti *et al*. [38] can also bias the estimations [38], while discrepancies can also be produced by either genotyping errors that inflate the Hulbert genome size estimator and/or the non-random clustering of markers such as AFLPs [54,55].

In the present study, a consensus map of maritime pine was generated from segregation data obtained in full-sib (G2) and selfed (F2) populations. The integrated map provided the relative position of 280 framework and unique SNPs, as well as additional 74 markers (SSRs, EST-Ps, candidate genes). The consensus map comprised 354 markers in total, with two adjacent markers spaced by 4 cM on average. With these parameters, we estimated, the coverage of maritime pine genome using the formula: *c *= 1-e^-2*dn*/*L *^[56], with *c *representing the proportion of the genome within *d *cM of a marker, where *L *is the estimated genome length and *n *is the number of markers. Assuming a random distribution of markers, a genome size of 2,500 cM and an average marker spacing of 4 cM, the estimated proportion of the maritime pine genome covered by our analysis was 67.8%. In order to evaluate the number of markers needed to cover 95% of the expected maritime pine genome (2,500 cM), we used an additional formula developed by Lange and Boehnke [56], i.e. *n *= [log(1-*p*)/log(1-2*c*/*k*)], where n is the minimum number of randomly distributed markers needed to cover a proportion *p*, of a genome size *k*, at a maximum distance of 2*c *between two adjacent markers. We estimated that 373 and 935 markers were necessary to cover 95% of the genome with a maximum distance of 20 or 4 cM between markers, respectively.

The consensus map was comprised of 24 linkage groups, while the haploid number of chromosomes for *Pinus *is 12. However, the 354 markers mapped are not far away from the number of markers expected to cover 95% of the genome for a maximum distance of 20 cM between markers. This suggests that the markers are probably not distributed randomly along the genome and that more markers should be needed to saturate the map. As suggested by Sewell *et al*. [55], the gaps between the split linkage groups belonging to the same unit may represent a non-random sampling of the genome resulting from an under-representation of markers from these regions. Li and Yeh [53] indicated that small pedigree size and low linkage information content can also cause undetected linkages when a small number of genetic markers are used. When a large number of markers are used, unresolved linkages can be observed because markers are clustered into large regions separated by several gaps. In the present study, the consensus map integrated linkage data of only 87 shared SNP loci from two mapping populations of about 90 offsprings, the 267 other SNPs segregating in only one pedigree. In order to fill in the gaps between split linkage groups and thus saturate the consensus map, we should increase the sample size (or the number of pedigrees) and the number of anchor markers between mapping populations.

### Comparative mapping between loblolly and maritime pines and resource development for comparative genomics in conifers

Comparative mapping consists of studying the conservation of gene content (synteny) and order (colinearity) in the genome of related species and allows inferring chromosome evolution and transferring genetic information between species. Comparisons are generally made by detecting orthologous loci through sequences homology or conserved map regions [57,58]. Loblolly pine has emerged as a reference species for comparative mapping in the Pinaceae [7,16]. Comparative mapping was already investigated between *P*. *taeda *and other *Pinus *species using putatively orthologous markers [17,58-60]. Macro-synteny was also reported between maritime and loblolly pines [6] and 32 shared EST-Ps made it possible to identify 10 homologous LGs. In the present study, we made an *a posteriori *search for additional orthologous loci between markers mapped on loblolly and maritime pines. We identified 50 putative COS markers of which 46 showed multiple parallel linkages between 7 linkage groups; the remaining 4 COS markers mapped in non orthologous linkage groups, thus suggesting that they belonged to different members of the same gene families. Altogether, 77 COS markers (31 EST-Ps and 46 SNPs) allowed alignment of all 12 homologous linkage groups between both pine species. Macro-synteny and macro-colinearity were generally well conserved, but some inconsistencies in the marker order within LGs were observed. Such inconsistencies can be attributed either to different members of gene families with different map positions within the same LG, or to the low resolution of the marker order in the consensus map, because of a low number of anchor loci. To ensure that future comparative mapping studies will rely on an *a priori *defined set of orthologous markers instead of defining orthology between mapped markers *a posteriori*, we have provided a set of COS markers between the two most economically important conifer genera *Pinus *and *Picea*.

## Conclusion

The present study demonstrates the usefulness of highly multiplexed SNP genotyping arrays to rapidly generate informative molecular markers showing Mendelian segregation in different mapping pedigrees and to establish the first SNP-based genetic linkage map in non-model species such as maritime pine. The genotyping of five other maritime pine mapping populations is underway and will allow us to create a more precise and denser consensus map for this species. The sequencing of parental lines of these mapping pedigrees has recently been carried out using 454-Roche sequencing to extend the set of informative SNPs (data available on the Short Read Archive of the NCBI). Crossing this extended set of SNPs to the COS markers identified in this study will provide new resources for high resolution comparative genomics at the dawn of the first conifer full-genome sequences.

## Authors' contributions

EC and CB sampled the plant material and extracted the DNA. AB checked the quality of the DNA. AJE, JW and DN provided most of the resequenced amplicons (CRSP project) and provided loblolly pine mapping information for comparative mapping. JPJC and SCGM provided plant material for CRSP project and checked the re-sequenced data. DG was involved in additional candidate gene resequencing. GLP, PGG and JMF were involved in generating the unigene set from maritime pine Sanger ESTs, and GLP wrote the associated section of the manuscript. GLP, PC and MTC were involved in cDNA and SSH library construction. CL coordinated and performed the SNP analysis from sequence analysis to array design with the help of PGG. CL wrote the associated sections of the manuscript. YCL, YvdP and PR carried out the COS analysis and wrote associated sections of the manuscript. DZ coordinated the genotyping work. EC and PC validated the polymorphic SNPs. EC constructed the linkage maps. CP and MTC coordinated the project, conceived and designed the experiment. AK coordinated the Evoltree Network of Excellence that provided the funding for the genotyping. EC and CP wrote the paper. All authors read and approved the final manuscript.

## Supplementary Material

Additional file 1 **cDNA libraries and sequencing**. Click here for file

Additional file 2 **List of the 49 candidate gene fragments used for *in vitro *SNP detection**. Click here for file

Additional file 3 **List of the 1,536 SNPs and associated features**. Click here for file

Additional file 4 **Summary of the different types of markers combined with SNP markers for the construction of the G2 linkage maps**. Click here for file

Additional file 5 **Genetic linkage maps for maritime pine: G2 Female and G2 Male, F2 and consensus**. Click here for file

Additional file 6 **Number of SNP markers and contigs mapped on the G2 and F2 linkage maps**. Click here for file

Additional file 7 **Number of orthologous markers mapped on the loblolly and maritime pine linkage groups**. Click here for file

Additional file 8 **COS markers between TGI pine, TGI spruce and maritime pine unigene**. Click here for file
