# Supplementary material for: Development and implementation of a highly-multiplexed SNP array for genetic mapping in maritime pine and comparative mapping with loblolly pine
Source: BMC Genomics. 2011 Jul 18;12:368. doi: 10.1186/1471-2164-12-368 (PMC3146957; doi:10.1186/1471-2164-12-368)
Supplement: Additional file 1 — cDNA libraries and sequencing. [file 1471-2164-12-368-S1.PDF]

Additional file 1: cDNA libraries and sequencing

Plant material

All libraries were comprised of a unique tissue collected on several genotype of maritime pine. ESTs were obtained from the following tissues:

cDNA libraries:

(i) Xylem library: Sampling of differentiating xylem samples was carried out in 1998, 1999, 2000 and 2001 on four adult maritime pine genotypes (accessions #105, #305, #4015 and #1906). A total of 14 samples were collected on straight and artificially bent trees (from 6 hours to 18 months) at the beginning (early wood) and at the end (late wood) of the growing season as shown in table 1. From the bent trees (15° away from the vertical position), samples were collected at the upper (opposite side) and lower (compression side) part of the trunk. Trees originated from a clonal test of Corsican provenances (test nº 6-44-7) planted in 1986 at the forestry station of INRA-Pierroton (France, Aquitaine).

	EW	LW	CW	OW	CW	OW	CW	OW	CW	OW	CW	OW	CW	OW
accession			6 hours	6 hours	1 day	1 day	8 day	8 day	40 days	40 days	120 days	120 days	18 months	18 months
1906			X	X										
4015					X	X								
105							X	X	X	X	X	X	X	X
305	X	X												

Table 1 -Origin of the differentiating xylem samples used for cDNA library construction. Abbreviations correspond to: EW: early wood, LW: late wood, CW: compression wood, OW: opposite wood.

(ii) Needle and (iii) root libraries: Briefly, maritime pine seeds were sterilized in 30% H_2O_2 for 15 min, before 24h imbibitions in sterile water and placed on sterile moistened filter paper. After 10 days of germination, 48 seedlings were transferred to an aerated nutrient solution. After three weeks of growth, a drought stress treatment was applied to half of the seedlings by lowering the osmotic potential of the nutrient solution to -0.45Mpa, using polyethylene glycol (PEG3350 Sigma-Adrich, France) as an osmoticum. The other half was kept in the nutrient solution (-0.08 MPa) and used as the control treatment. Roots and aerial parts were harvested separately, frozen immediately in liquid nitrogen, and stored at –80°C for RNA isolation.
(iv) Bud library: a specific library was constructed using RNA extracted from buds collected on three Spanish genotypes at different developmental stages. This work was done at INIA research station (Madrid, Spain)

Subtractive Suppressive Libraries (SSH)

The following tissues were used:
(i) Identification of "seasonal" responsive genes: We used the previously described clonal test to harvest differentiating xylem at the beginning (EW) and at the end (LW) of the growing season on 2 maritime pine accessions (#4015 and #3006).

(ii) Identification of "ontogenic" responsive genes: Juvenile (JW) and mature (MW) differentiating xylem samples were harvested in 2006 at INRA research station. We used two 30 years old genotypes to sample differentiating xylem at the base (MW) and at the top (JW) of the tree.

(iii) Identification of genes specifically expressed in differentiating xylem: Two pools of RNA were used. Pool #1 was obtained by mixing equal amount of total RNA extracted from differentiating xylem associated to EW, LW, MW and JW, while pool#2 was obtained by mixing equal amount of total RNA extracted from needles, buds, phloem and cambium harvested on 2 maritime pine accessions (#4015 and #3006).

RNA extraction and cDNA libraries construction

cDNA libraries

Total RNA corresponding to differentiating xylems, buds, roots and needles was extracted according to Chang et al.(1993) with a final purification using the RNAeasy kit (QIAGEN, Courtaboeuf, France). PolyA RNA was isolated using Oligotex kit (QIAGEN). cDNA were synthetized using the Stratagene cDNA synthesis kit (Strategene, La Jolla, CA, USA) with the following modifications: no radioactivity was used. Following the reverse transcription step, 1 µL of AMV (*avian myeloblastosis* virus) was added and samples were incubated for 1h at 42°C. Size fractionation of cDNAs was then carried out using SizeSep 400 columns (Amersham Biosciences, Freiburg, Germany) and phenol extraction after size fractionation was therefore avoided. The resulting cDNAs were packaged into λ ZAP II phages using the Gigapack III Gold packaging kit (Strategene, La Jolla, CA, USA).
Differentiating xylem, roots and needles clones were finally excised to generate plasmid clones. Colonies were randomly picked and clones were arranged individually in 96 -well microtiter plates for storage and processing. Individual plasmid clones were grown overnight at 37ºC in wells containing 100µL of LB-ampicillin and stored at –80°C with glycerol up to 25%.

Subtractive Suppressive Libraries (SSH)

Total RNA was extracted according to the protocol described by Le Provost et al. (2007). SSH was then performed using the Smart PCR synthesis and the PCR select cDNA substration kits ccording to the manufacturer's instructions. Amplified, differentially expressed cDNA fragments were then cloned into the pGEM T easy vector (Promega, Madisson, USA). For Each SSH library, 1,536 clones were picked and subjected to 5' single pass sequencing. The sequencing was done at "Centre National de Sequençage". ESTs were obtained from the following SSH libraries: (i) four SSH libraries were built by subtracting RNA from differentiating xylem associated to EW vs. LW, JW vs. MW and vice versa, (ii) an additional library was built for xylem specific genes by subtracting RNA from pool#1 (differentiating xylem) to pool#2 (other tissues).

Sequencing

Clones were randomly isolated and arranged individually in 384 -well microtiter plates for storage and processing and subjected to high-throughput single-path sequencing from their 5'

ends using the classical sanger sequencing technique. Bud and SSH libraries, were sequenced at “Centre National de Sequençage” (Evry, France).
Sequences for the remaining libraries were obtained at the Genome-Transcriptome platform located in Bordeaux (http://www.pierroton.inra.fr/biogeco/site_pole_agro/genoseq.html)

Suppressive Subtractive Hybridization and non normalized cDNA libraries were derived from several genotypes of maritime pine, representing either different tissues (differentiating xylem, roots, bud and needles) or different experimental treatments (i.e. drought stressed plants). A total of 40,774 sequencing runs were performed comprising 7,680 clones and 33,094 clones from SSH (1,536 clones per library) and cDNA libraries respectively. In detail, for cDNA libraries, 9,814, 12,963, 9,035 and 1,282 clones were respectively picked up from differentiating xylem(library F) , buds (library G), roots (library H) and needles (library I) library and then subjected to high-throughput single-path sequencing from their 5' ends (Table 2). After vector and low complexity area masking, all sequences shorter than 60 bp were removed from the analysis. Finally, unexploitable chromatogramms and quality checking lead us to eliminate 9,096 sequences (22%) resulting in 31,678 high quality sequences with an average length of 487 nucleotides and counting 8,1Mb of bases with a quality score greater or equal to 20 (99% accuracy per base according to *Phred* definition) were obtained and were used in the assembly step.

Tissue	Genotypes	Library Short name	Number of clones sequenced	Number of informative reads	Average length (bp)	Sequencing success rate	Gene discovery rate	Redundancy
				**Subtractive Suppressive libraries (SSH)**				
Mature wood enriched library	2 landes provenances	A	1,536	1,269	430	80%	34%	40%
Juvenile wood enriched library	2 landes provenances	B	1,536	1,143	479	72%	39%	16%
Earlywood enriched library	2 corsican provenances	C	1,536	1,113	418	70%	35%	39%
Latewood enriched library	2 corsican provenances	D	1,536	1,220	427	79%	28%	54%
Differentiating xylem enriched library	2 corsican provenances	E	1,536	1,215	393	79%	49%	53%
				**Conventional cDNA libraries**				
Differentiating xylem	4 corsican provenances	F	9,814	7,905	524	80%	38%	44%
Buds	3 spanish provenances	G	9,035	8,903	598	98%	52%	37%
Roots	Medocan natural stands (48 individuals)	H	12,963	8,203	438	63%	48%	50%
Needles	Medocan natural stands (48 individuals)	I	1,282	707	530	55%	32%	34%
Total			**40,774**	**31,678**	**487**	**77%**	**NA**	**56%**

Table 2: overview of the EST data set. The average length was calculated using only the singletons data available for each SSH or cDNA library.

Chang S, Puryear J, Cairney J (1993) A simple and efficient method for isolating RNA from pine trees. Plant Mol. Biol. Rep. 11: 113-116

Le Provost G, Herrera R, Paiva J, Chaumeil P, Salin F, Plomion C (2007) A micromethod for high throughput RNA extraction in forest trees. Biological Research 40(3), 291-297
