# Supplementary material for: Development and implementation of a highly-multiplexed SNP array for genetic mapping in maritime pine and comparative mapping with loblolly pine
Source: BMC Genomics. 2011 Jul 18;12:368. doi: 10.1186/1471-2164-12-368 (PMC3146957; doi:10.1186/1471-2164-12-368)
Supplement: Additional file 4 — Summary of the different types of markers combined with SNP markers for the construction of the G2 linkage maps. [file 1471-2164-12-368-S4.PDF]

Additional file 4: **Summary of the different types of markers combined with SNP markers for the construction of the G2 linkage maps**.

Type of markers	AFLP	SSR	EST-P	Candidate genes
Number of markers	530	28	50	11
Segregation type	1 : 1 (271 ♀ and 259 ♂)	1 : 1 (6 ♀ and 7 ♂)<br>1 :2: 1 (4)<br>1 :1: 1: 1 (11)	1 : 1 (24 ♀ and 11 ♂)<br>1 :2: 1 (9)<br>1 :1: 1: 1 (6)	1 : 1 (1 ♂)<br>1 :2: 1 (4)<br>1 :1 :1 :1 (6)
Number of offsprings	201	90	90	46
References	Chagné *et al.* 2002	Chagné *et al.* 2004	Chagné *et al.* 2003	Pot *et al.* 2006

Abbreviations are as follows: AFLP; Amplified fragment-length polymorphism, SSR; simple sequence repeat, EST-P*;* Expressed Sequence Tags Polymorphism.

References cited:

Chagné D, Lalanne C, Madur D, Kumar S, Frigério J-M, Krier C, Decroocq S, Savouré[c], Magida Bou-Dagher-Kharrat A, Bertocchi E, Brach J and Plomion C: **A high density genetic map of maritime pine based on AFLPs.** *Ann. For. Sci.* 2002, **59**: 627-636

Chagné D, Brown G, Lalanne C, Madur D, Pot D, Neale D and Plomion C: **Comparative genome and QTL mapping between maritime and loblolly pines.** *Molecular Breeding,* 2003, **12:** 185–195

Chagné D, Chaumeil P, Ramboer A, Collada C, Guevara A, Cervera MT, Vendramin GG, Garcia V, Frigerio JM, Echt C, Richardson T, Plomion C: **Cross-species transferability and mapping of genomic and cDNA SSRs in pines.** Theor Appl Genet. 2004, 109:1204-1214

Pot D, Rodrigues JC, Rozenberg P, Chantre G, Tibbits J, Cahalan C, Pichavant Fand Plomion C: **QTLs and candidate genes for wood properties in maritime pine (*Pinus pinaster* Ait.)** *Tree Genetics & Genomes*, 2006, **2**: 10-24
