# Supplementary material for: Development and implementation of a highly-multiplexed SNP array for genetic mapping in maritime pine and comparative mapping with loblolly pine
Source: BMC Genomics. 2011 Jul 18;12:368. doi: 10.1186/1471-2164-12-368 (PMC3146957; doi:10.1186/1471-2164-12-368)
Supplement: Additional file 5 — Genetic linkage maps for maritime pine: G2 Female and G2 Male, F2 and consensus. [file 1471-2164-12-368-S5.PDF]

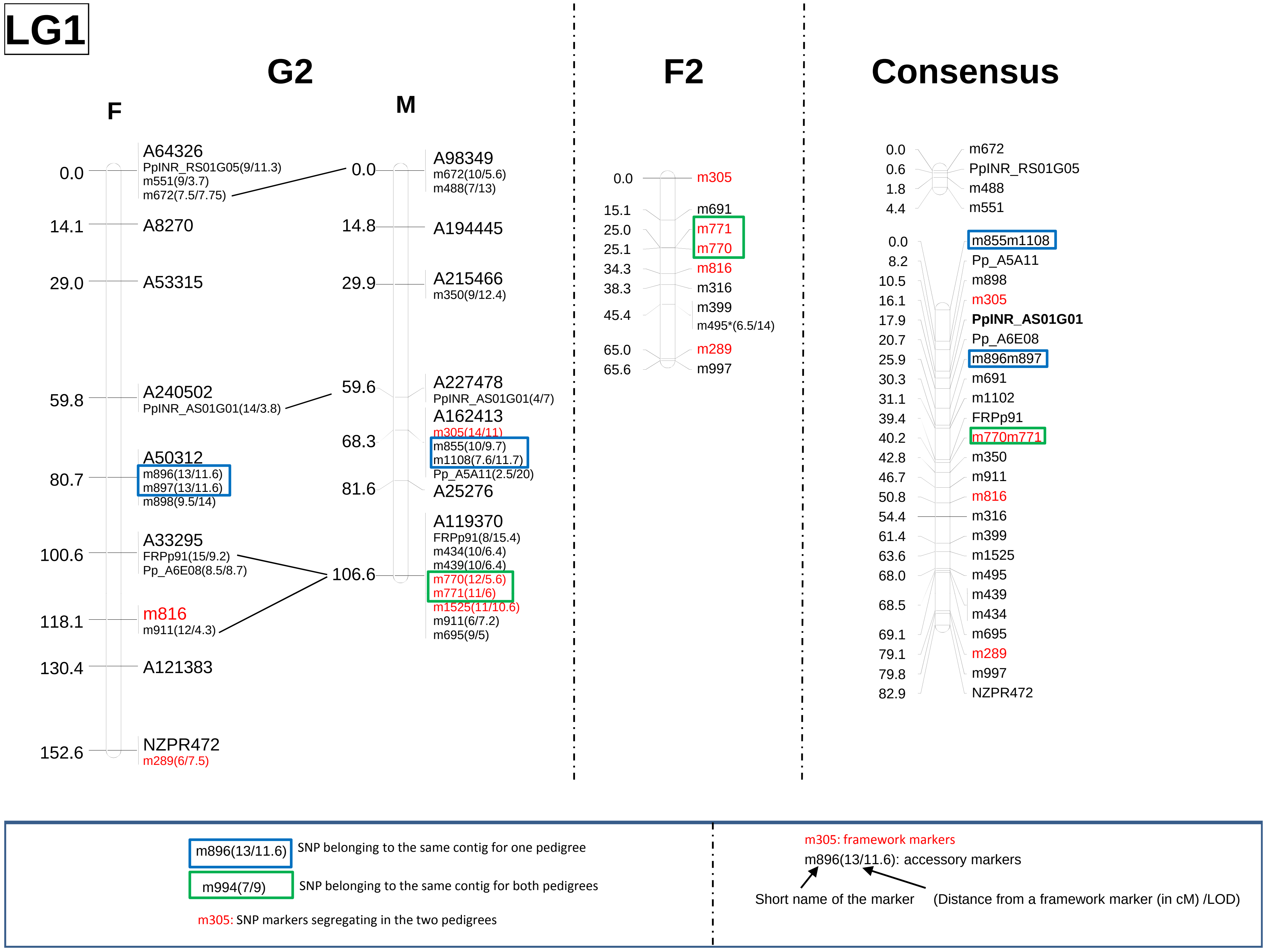

LG1
G2
F
M
F2
Consensus
0.0 A64326
PpINR_RS01G05(9/11.3)
m551(9/3.7)
m672(7.5/7.75)
14.1 A8270
29.0 A53315
59.8 A240502
PpINR_AS01G01(14/3.8)
80.7 A50312
m896(13/11.6)
m897(13/11.6)
m898(9.5/14)
100.6 A33295
FRPp91(15/9.2)
Pp_A6E08(8.5/8.7)
118.1 m816
m911(12/4.3)
130.4 A121383
152.6 NZPR472
m289(6/7.5)
0.0 A98349
m672(10/5.6)
m488(7/13)
14.8 A194445
29.9 A215466
m350(9/12.4)
59.6 A227478
PpINR_AS01G01(4/7)
A162413
68.3 m305(14/11)
m855(10/9.7)
m1108(7.6/11.7)
Pp_A5A11(2.5/20)
81.6 A25276
A119370
FRPp91(8/15.4)
m434(10/6.4)
m439(10/6.4)
106.6 m770(12/5.6)
m771(11/6)
m1525(11/10.6)
m911(6/7.2)
m695(9/5)
0.0 m305
15.1 m691
25.0 m771
25.1 m770
34.3 m816
38.3 m316
45.4 m399
m495*(6.5/14)
65.0 m289
65.6 m997
0.0 m672
0.6 PpINR_RS01G05
1.8 m488
4.4 m551
0.0 m855m1108
8.2 Pp_A5A11
10.5 m898
16.1 m305
17.9 PpINR_AS01G01
20.7 Pp_A6E08
25.9 m896m897
30.3 m691
31.1 m1102
39.4 FRPp91
40.2 m770m771
42.8 m350
46.7 m911
50.8 m816
54.4 m316
61.4 m399
63.6 m1525
68.0 m495
68.5 m439
m434
69.1 m695
79.1 m289
79.8 m997
82.9 NZPR472
m896(13/11.6) SNP belonging to the same contig for one pedigree
m994(7/9) SNP belonging to the same contig for both pedigrees
m305: SNP markers segregating in the two pedigrees
m305: framework markers
m896(13/11.6): accessory markers
Short name of the marker
(Distance from a framework marker (in cM) /LOD)

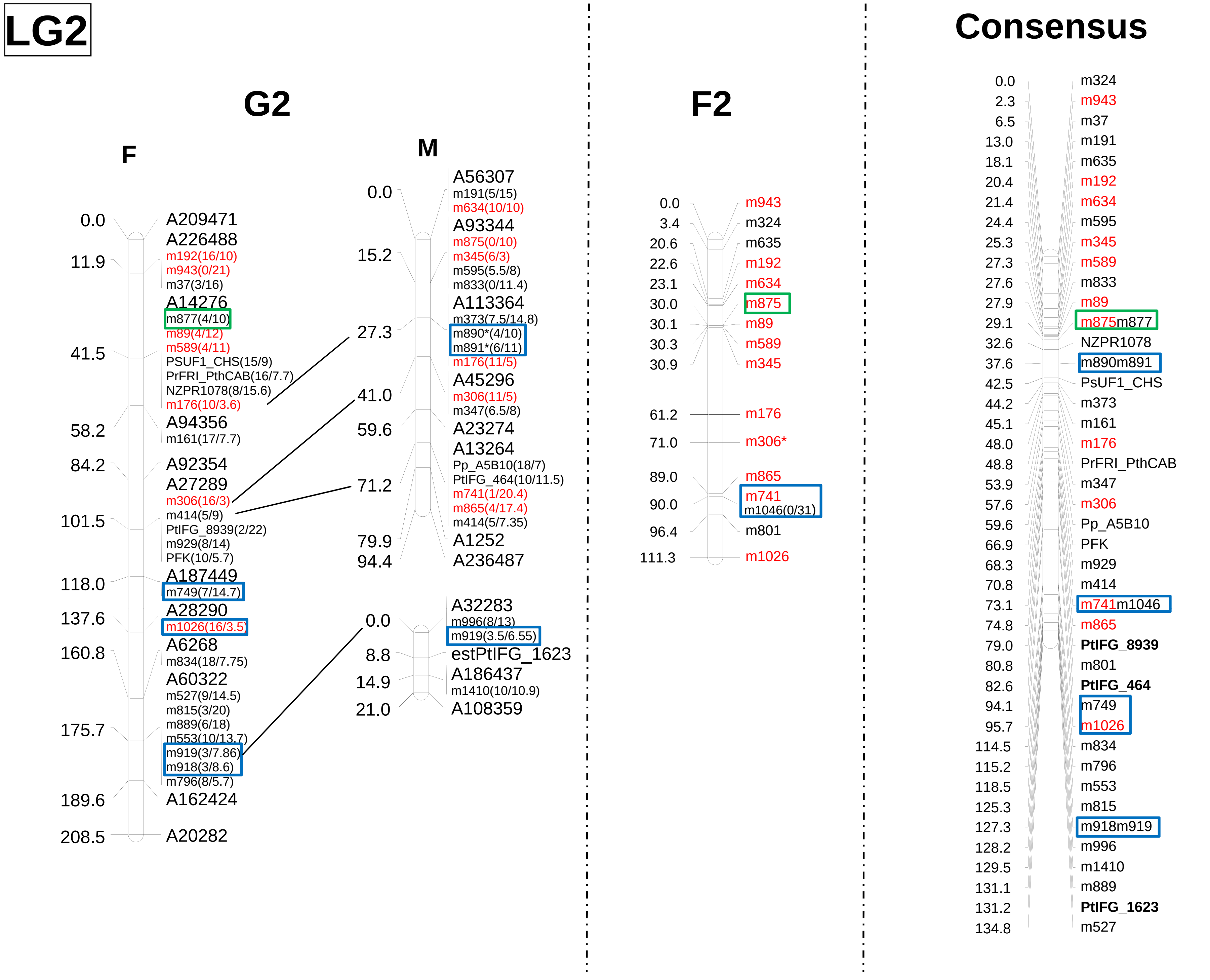

LG2
G2
F
M
F2
Consensus
A209471
A226488
m192(16/10)
m943(0/21)
m37(3/16)
A14276
m877(4/10)
m89(4/12)
m589(4/11)
PSUF1_CHS(15/9)
PrFRI_PthCAB(16/7.7)
NZPR1078(8/15.6)
m176(10/3.6)
A94356
m161(17/7.7)
A92354
A27289
m306(16/3)
m414(5/9)
PtIFG_8939(2/22)
m929(8/14)
PFK(10/5.7)
A187449
m749(7/14.7)
A28290
m1026(16/3.5)
A6268
m834(18/7.75)
A60322
m527(9/14.5)
m815(3/20)
m889(6/18)
m553(10/13.7)
m919(3/7.86)
m918(3/8.6)
m796(8/5.7)
A162424
A20282
A56307
m191(5/15)
m634(10/10)
A93344
m875(0/10)
m345(6/3)
m595(5.5/8)
m833(0/11.4)
A113364
m373(7.5/14.8)
m890*(4/10)
m891*(6/11)
m176(11/5)
A45296
m306(11/5)
m347(6.5/8)
A23274
A13264
Pp_A5B10(18/7)
PtIFG_464(10/11.5)
m741(1/20.4)
m865(4/17.4)
m414(5/7.35)
A1252
A236487
A32283
m996(8/13)
m919(3.5/6.55)
estPtIFG_1623
A186437
m1410(10/10.9)
A108359
m943
m324
m635
m192
m634
m875
m89
m589
m345
m176
m306*
m865
m741
m1046(0/31)
m801
m1026
m324
m943
m37
m191
m635
m192
m634
m595
m345
m589
m833
m89
m875m877
NZPR1078
m890m891
PsUF1_CHS
m373
m161
m176
PrFRI_PthCAB
m347
m306
Pp_A5B10
PFK
m929
m414
m741m1046
m865
PtIFG_8939
m801
PtIFG_464
m749
m1026
m834
m796
m553
m815
m918m919
m996
m1410
m889
PtIFG_1623
m527

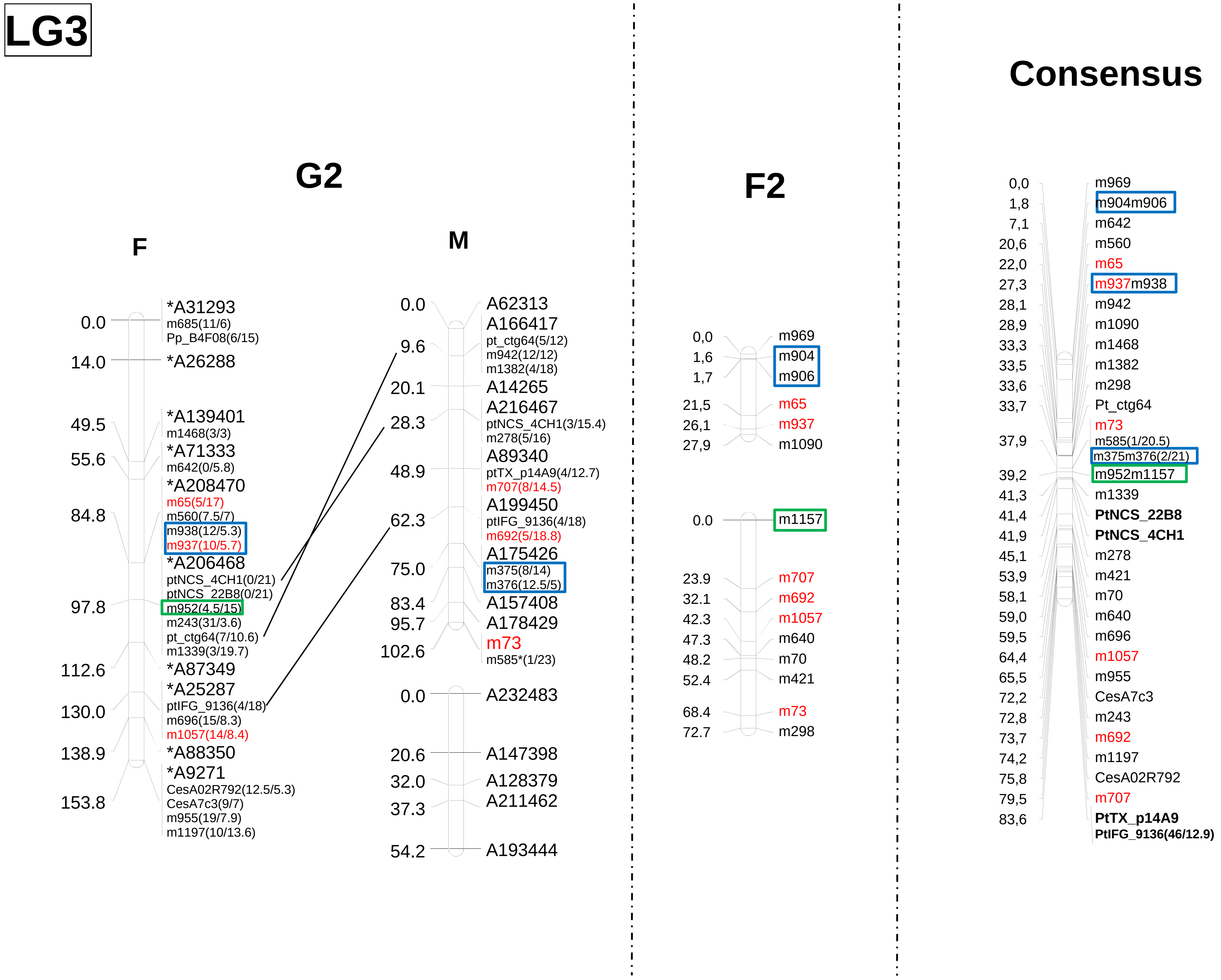
LG3
G2
F
M
F2
Consensus
*A31293
m685(11/6)
Pp_B4F08(6/15)
*A26288
*A139401
m1468(3/3)
*A71333
m642(0/5.8)
*A208470
m65(5/17)
m560(7.5/7)
m938(12/5.3)
m937(10/5.7)
*A206468
ptNCS_4CH1(0/21)
ptNCS_22B8(0/21)
m952(4.5/15)
m243(31/3.6)
pt_ctg64(7/10.6)
m1339(3/19.7)
*A87349
*A25287
ptIFG_9136(4/18)
m696(15/8.3)
m1057(14/8.4)
*A88350
*A9271
CesA02R792(12.5/5.3)
CesA7c3(9/7)
m955(19/7.9)
m1197(10/13.6)
A62313
A166417
pt_ctg64(5/12)
m942(12/12)
m1382(4/18)
A14265
A216467
ptNCS_4CH1(3/15.4)
m278(5/16)
A89340
ptTX_p14A9(4/12.7)
m707(8/14.5)
A199450
ptIFG_9136(4/18)
m692(5/18.8)
A175426
m375(8/14)
m376(12.5/5)
A157408
A178429
m73
m585*(1/23)
A232483
A147398
A128379
A211462
A193444
m969
m904
m906
m65
m937
m1090
m1157
m707
m692
m1057
m640
m70
m421
m73
m298
m969
m904m906
m642
m560
m65
m937m938
m942
m1090
m1468
m1382
m298
Pt_ctg64
m73
m585(1/20.5)
m375m376(2/21)
m952m1157
m1339
PtNCS_22B8
PtNCS_4CH1
m278
m421
m70
m640
m696
m1057
m955
CesA7c3
m243
m692
m1197
CesA02R792
m707
PtTX_p14A9
PtIFG_9136(46/12.9)

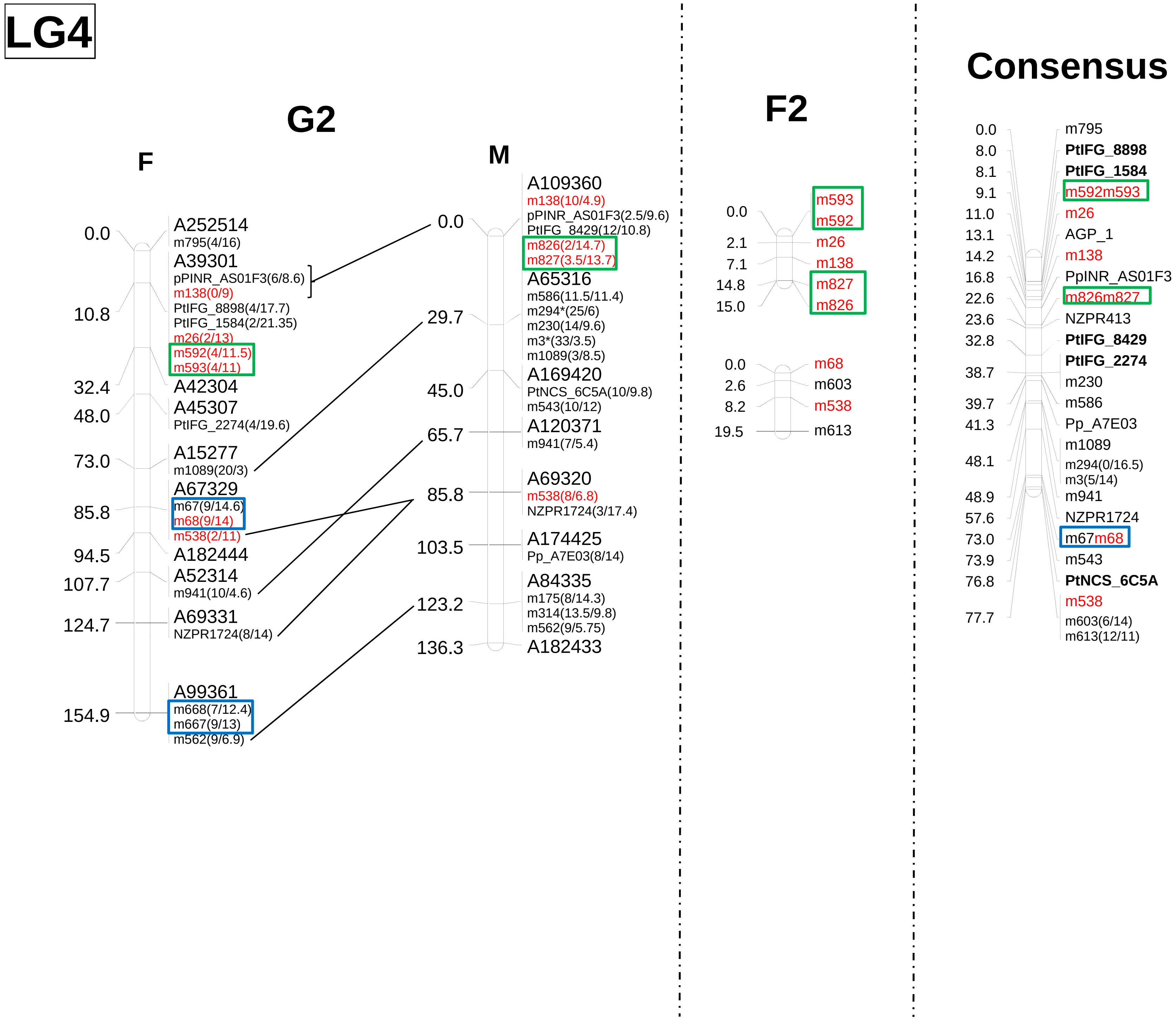
LG4
G2
F
M
F2
Consensus
0.0 A252514 m795(4/16)
A39301 pPINR_AS01F3(6/8.6) m138(0/9)
10.8 PtIFG_8898(4/17.7) PtIFG_1584(2/21.35) m26(2/13)
m592(4/11.5) m593(4/11)
32.4 A42304
48.0 A45307 PtIFG_2274(4/19.6)
73.0 A15277 m1089(20/3)
85.8 A67329 m67(9/14.6) m68(9/14) m538(2/11)
94.5 A182444
107.7 A52314 m941(10/4.6)
124.7 A69331 NZPR1724(8/14)
154.9 A99361 m668(7/12.4) m667(9/13) m562(9/6.9)
0.0 A109360 m138(10/4.9) pPINR_AS01F3(2.5/9.6) PtIFG_8429(12/10.8) m826(2/14.7) m827(3.5/13.7)
29.7 A65316 m586(11.5/11.4) m294*(25/6) m230(14/9.6) m3*(33/3.5) m1089(3/8.5)
45.0 A169420 PtNCS_6C5A(10/9.8) m543(10/12)
65.7 A120371 m941(7/5.4)
85.8 A69320 m538(8/6.8) NZPR1724(3/17.4)
103.5 A174425 Pp_A7E03(8/14)
123.2 A84335 m175(8/14.3) m314(13.5/9.8) m562(9/5.75)
136.3 A182433
0.0 m593 m592
2.1 m26
7.1 m138
14.8 m827
15.0 m826
0.0 m68
2.6 m603
8.2 m538
19.5 m613
0.0 m795
8.0 PtIFG_8898
8.1 PtIFG_1584
9.1 m592m593
11.0 m26
13.1 AGP_1
14.2 m138
16.8 PpINR_AS01F3
22.6 m826m827
23.6 NZPR413
32.8 PtIFG_8429
38.7 PtIFG_2274
39.7 m230
41.3 m586
48.1 Pp_A7E03
48.9 m1089
57.6 m294(0/16.5) m3(5/14)
73.0 m941
73.9 NZPR1724
76.8 m67m68
77.7 m543
PtNCS_6C5A
m538
m603(6/14)
m613(12/11)

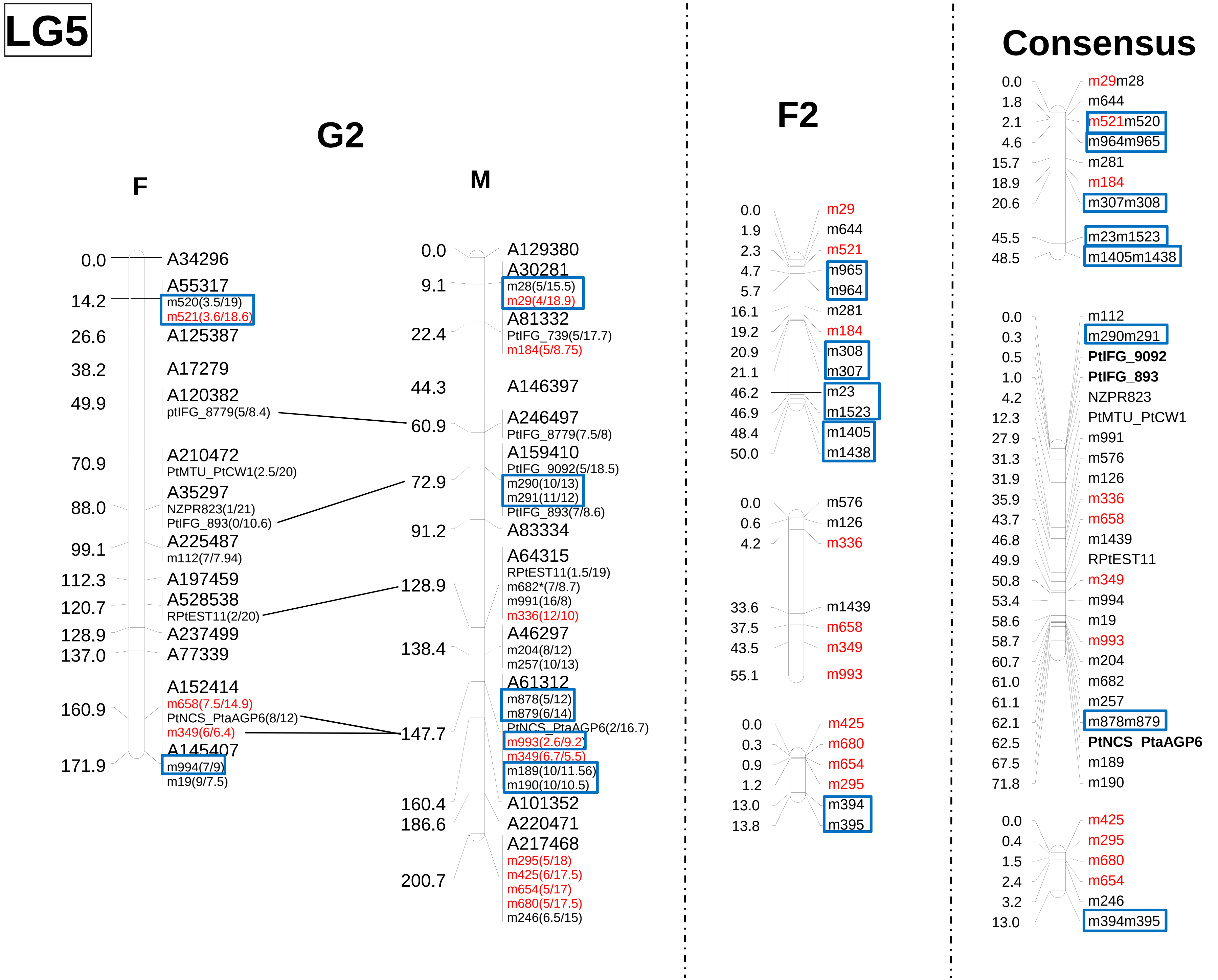

LG5
G2
F
M
F2
Consensus
A34296
A55317
m520(3.5/19)
m521(3.6/18.6)
A125387
A17279
A120382
ptIFG_8779(5/8.4)
A210472
PtMTU_PtCW1(2.5/20)
A35297
NZPR823(1/21)
PtIFG_893(0/10.6)
A225487
m112(7/7.94)
A197459
A528538
RPtEST11(2/20)
A237499
A77339
A152414
m658(7.5/14.9)
PtNCS_PtaAGP6(8/12)
m349(6/6.4)
A145407
m994(7/9)
m19(9/7.5)
A129380
A30281
m28(5/15.5)
m29(4/18.9)
A81332
PtIFG_739(5/17.7)
m184(5/8.75)
A146397
A246497
PtIFG_8779(7.5/8)
A159410
PtIFG_9092(5/18.5)
m290(10/13)
m291(11/12)
PtIFG_893(7/8.6)
A83334
A64315
RPtEST11(1.5/19)
m682*(7/8.7)
m991(16/8)
m336(12/10)
A46297
m204(8/12)
m257(10/13)
A61312
m878(5/12)
m879(6/14)
PtNCS_PtaAGP6(2/16.7)
m993(2.6/9.2)
m349(6.7/5.5)
m189(10/11.56)
m190(10/10.5)
A101352
A220471
A217468
m295(5/18)
m425(6/17.5)
m654(5/17)
m680(5/17.5)
m246(6.5/15)
m29
m644
m521
m965
m964
m281
m184
m308
m307
m23
m1523
m1405
m1438
m576
m126
m336
m1439
m658
m349
m993
m425
m680
m654
m295
m394
m395
m29m28
m644
m521m520
m964m965
m281
m184
m307m308
m23m1523
m1405m1438
m112
m290m291
PtIFG_9092
PtIFG_893
NZPR823
PtMTU_PtCW1
m991
m576
m126
m336
m658
m1439
RPtEST11
m349
m994
m19
m993
m204
m682
m257
m878m879
PtNCS_PtaAGP6
m189
m190
m425
m295
m680
m654
m246
m394m395

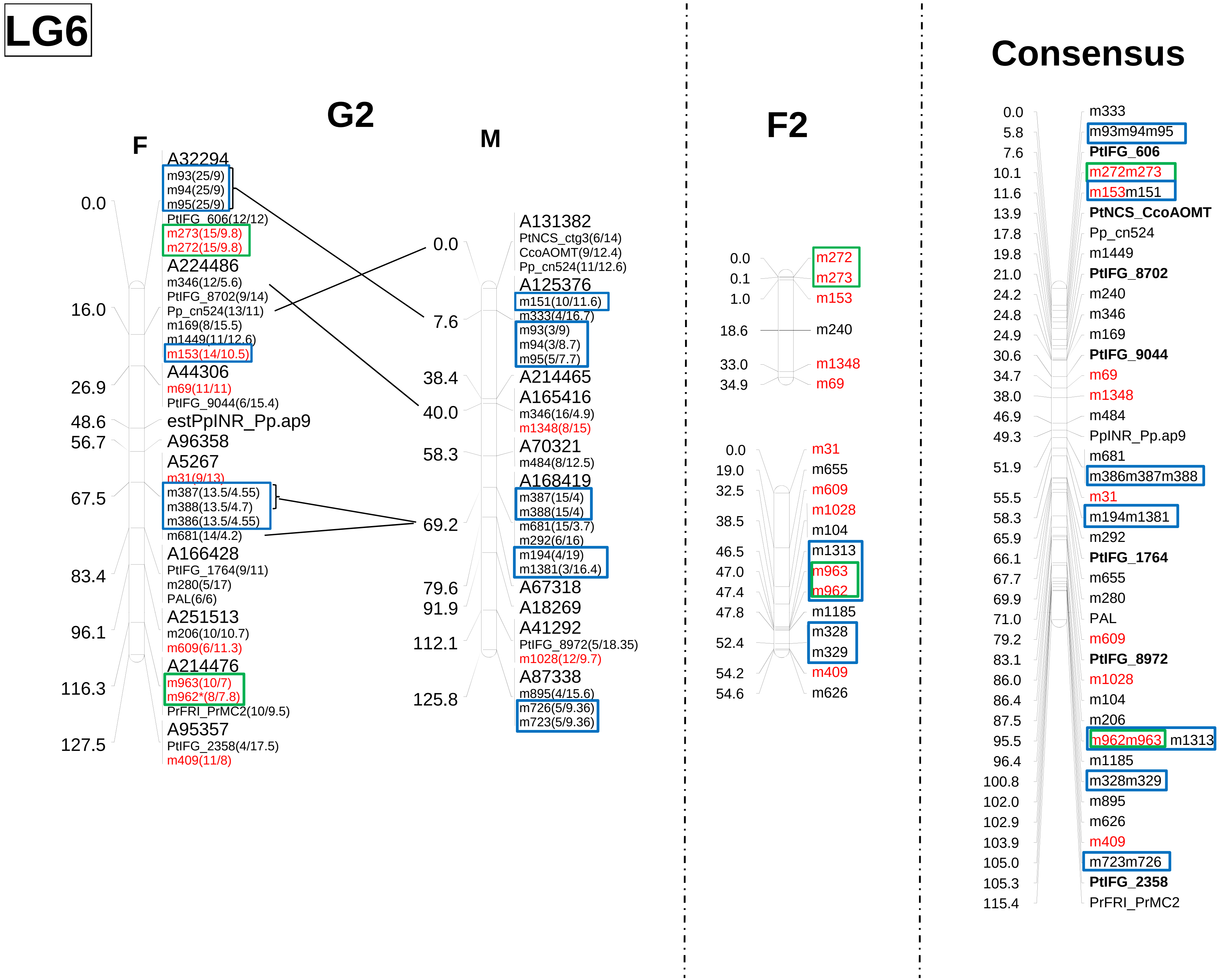
LG6
G2
F
M
F2
Consensus
0.0 A32294
m93(25/9)
m94(25/9)
m95(25/9)
PtIFG_606(12/12)
m273(15/9.8)
m272(15/9.8)
A224486
m346(12/5.6)
16.0 PtIFG_8702(9/14)
Pp_cn524(13/11)
m169(8/15.5)
m1449(11/12.6)
m153(14/10.5)
26.9 A44306
m69(11/11)
PtIFG_9044(6/15.4)
48.6 estPpINR_Pp.ap9
56.7 A96358
A5267
m31(9/13)
67.5 m387(13.5/4.55)
m388(13.5/4.7)
m386(13.5/4.55)
m681(14/4.2)
A166428
83.4 PtIFG_1764(9/11)
m280(5/17)
PAL(6/6)
96.1 A251513
m206(10/10.7)
m609(6/11.3)
A214476
116.3 m963(10/7)
m962*(8/7.8)
PrFRI_PrMC2(10/9.5)
127.5 A95357
PtIFG_2358(4/17.5)
m409(11/8)
0.0 A131382
PtNCS_ctg3(6/14)
CcoAOMT(9/12.4)
Pp_cn524(11/12.6)
A125376
7.6 m151(10/11.6)
m333(4/16.7)
m93(3/9)
m94(3/8.7)
m95(5/7.7)
38.4 A214465
40.0 A165416
m346(16/4.9)
m1348(8/15)
58.3 A70321
m484(8/12.5)
A168419
69.2 m387(15/4)
m388(15/4)
m681(15/3.7)
m292(6/16)
m194(4/19)
m1381(3/16.4)
79.6 A67318
91.9 A18269
112.1 A41292
PtIFG_8972(5/18.35)
m1028(12/9.7)
125.8 A87338
m895(4/15.6)
m726(5/9.36)
m723(5/9.36)
0.0 m272
0.1 m273
1.0 m153
18.6 m240
33.0 m1348
34.9 m69
0.0 m31
19.0 m655
32.5 m609
38.5 m1028
m104
46.5 m1313
47.0 m963
47.4 m962
47.8 m1185
52.4 m328
m329
54.2 m409
54.6 m626
0.0 m333
5.8 m93m94m95
7.6 PtIFG_606
10.1 m272m273
11.6 m153m151
13.9 PtNCS_CcoAOMT
17.8 Pp_cn524
19.8 m1449
21.0 PtIFG_8702
24.2 m240
24.8 m346
24.9 m169
30.6 PtIFG_9044
34.7 m69
38.0 m1348
46.9 m484
49.3 PpINR_Pp.ap9
m681
51.9 m386m387m388
55.5 m31
58.3 m194m1381
65.9 m292
66.1 PtIFG_1764
67.7 m655
69.9 m280
71.0 PAL
79.2 m609
83.1 PtIFG_8972
86.0 m1028
86.4 m104
87.5 m206
95.5 m962m963 m1313
96.4 m1185
100.8 m328m329
102.0 m895
102.9 m626
103.9 m409
105.0 m723m726
105.3 PtIFG_2358
115.4 PrFRI_PrMC2

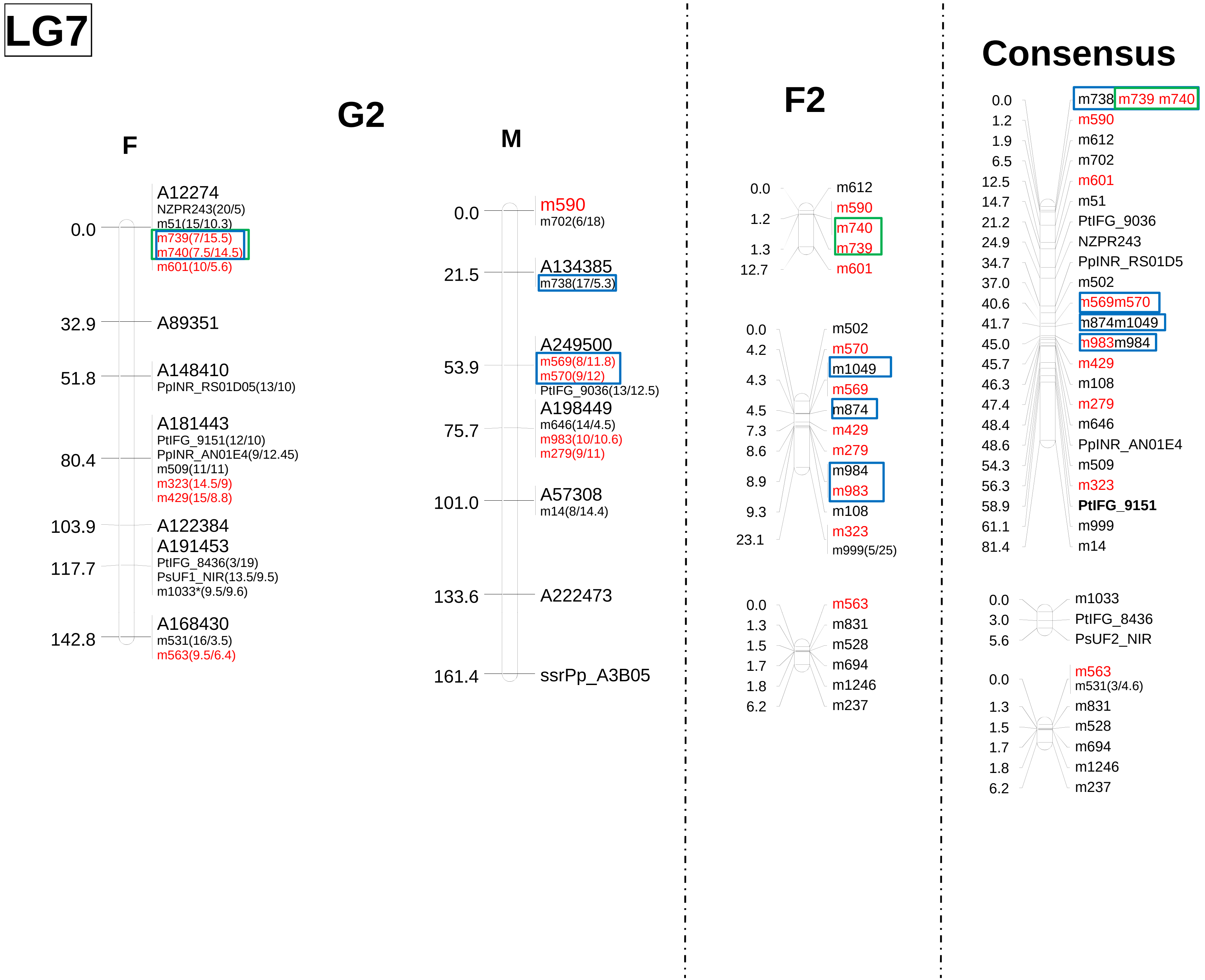
LG7
G2
F
0.0
A12274
NZPR243(20/5)
m51(15/10.3)
m739(7/15.5)
m740(7.5/14.5)
m601(10/5.6)
32.9
A89351
51.8
A148410
PpINR_RS01D05(13/10)
80.4
A181443
PtIFG_9151(12/10)
PpINR_AN01E4(9/12.45)
m509(11/11)
m323(14.5/9)
m429(15/8.8)
103.9
A122384
117.7
A191453
PtIFG_8436(3/19)
PsUF1_NIR(13.5/9.5)
m1033*(9.5/9.6)
142.8
A168430
m531(16/3.5)
m563(9.5/6.4)
M
0.0
m590
m702(6/18)
21.5
A134385
m738(17/5.3)
53.9
A249500
m569(8/11.8)
m570(9/12)
PtIFG_9036(13/12.5)
75.7
A198449
m646(14/4.5)
m983(10/10.6)
m279(9/11)
101.0
A57308
m14(8/14.4)
133.6
A222473
161.4
ssrPp_A3B05
F2
0.0 m612
1.2 m590
1.3 m740
12.7 m739
m601
0.0 m502
4.2 m570
4.3 m1049
4.5 m569
7.3 m874
8.6 m429
8.9 m279
9.3 m984
23.1 m983
m108
m323
m999(5/25)
0.0 m563
1.3 m831
1.5 m528
1.7 m694
1.8 m1246
6.2 m237
Consensus
0.0 m738 m739 m740
1.2 m590
1.9 m612
6.5 m702
12.5 m601
14.7 m51
21.2 PtIFG_9036
24.9 NZPR243
34.7 PpINR_RS01D5
37.0 m502
40.6 m569m570
41.7 m874m1049
45.0 m983m984
45.7 m429
46.3 m108
47.4 m279
48.4 m646
48.6 PpINR_AN01E4
54.3 m509
56.3 m323
58.9 PtIFG_9151
61.1 m999
81.4 m14
0.0 m1033
3.0 PtIFG_8436
5.6 PsUF2_NIR
0.0 m563
m531(3/4.6)
1.3 m831
1.5 m528
1.7 m694
1.8 m1246
6.2 m237

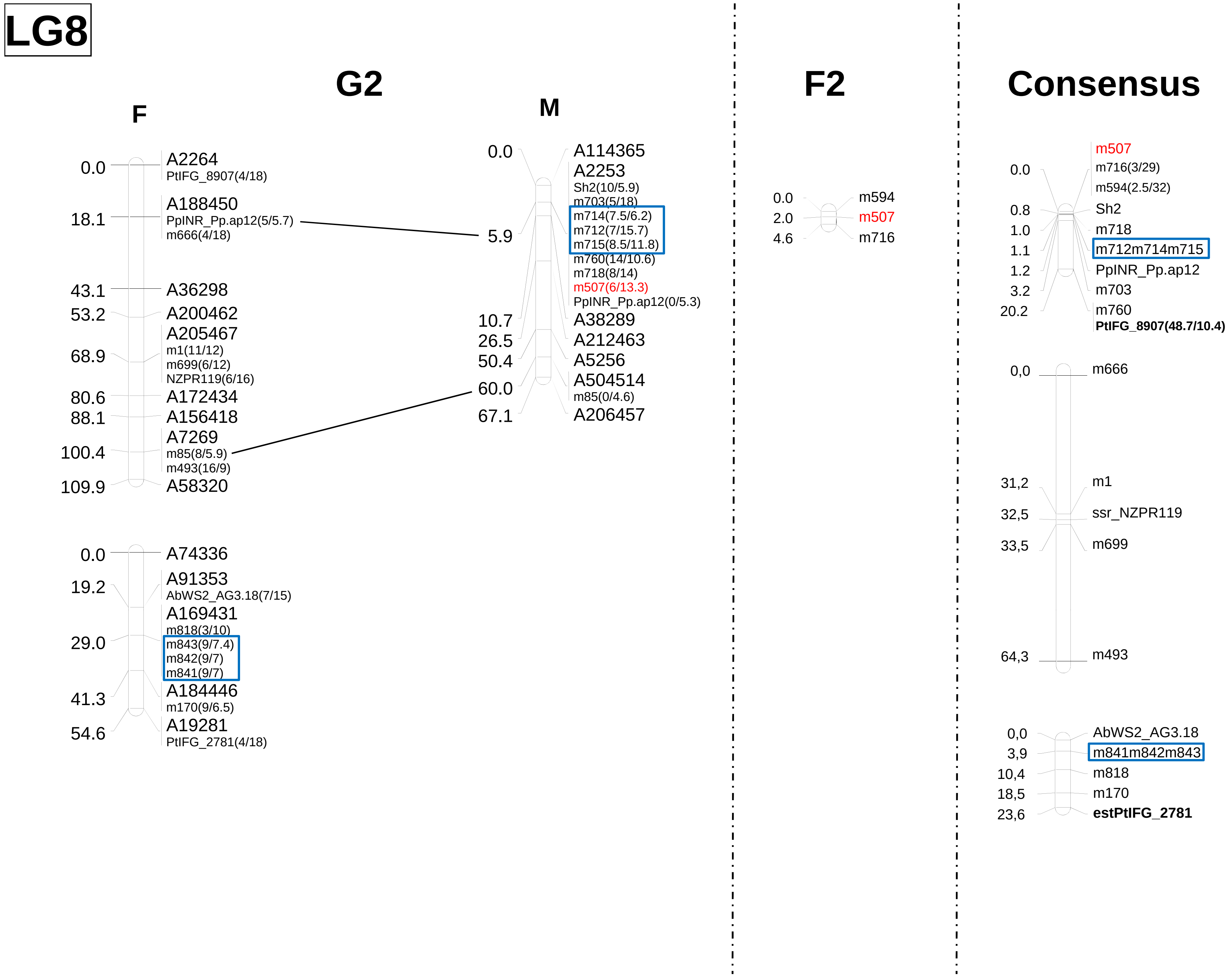
LG8
G2
F
M
F2
Consensus
0.0 A2264 PtIFG_8907(4/18)
18.1 A188450 PpINR_Pp.ap12(5/5.7) m666(4/18)
43.1 A36298
53.2 A200462
68.9 A205467 m1(11/12) m699(6/12) NZPR119(6/16)
80.6 A172434
88.1 A156418
100.4 A7269 m85(8/5.9) m493(16/9)
109.9 A58320
0.0 A74336
19.2 A91353 AbWS2_AG3.18(7/15)
29.0 A169431 m818(3/10) m843(9/7.4) m842(9/7) m841(9/7)
41.3 A184446 m170(9/6.5)
54.6 A19281 PtIFG_2781(4/18)
0.0 A114365 A2253 Sh2(10/5.9) m703(5/18)
5.9 m714(7.5/6.2) m712(7/15.7) m715(8.5/11.8) m760(14/10.6) m718(8/14) m507(6/13.3) PpINR_Pp.ap12(0/5.3)
10.7 A38289
26.5 A212463
50.4 A5256
60.0 A504514 m85(0/4.6)
67.1 A206457
0.0 m594
2.0 m507
4.6 m716
m507 m716(3/29) m594(2.5/32)
0.0
0.8 Sh2
1.0 m718
1.1 m712m714m715
1.2 PpINR_Pp.ap12
3.2 m703
20.2 m760 PtIFG_8907(48.7/10.4)
0,0 m666
31,2 m1
32,5 ssr_NZPR119
33,5 m699
64,3 m493
0,0 AbWS2_AG3.18
3,9 m841m842m843
10,4 m818
18,5 m170
23,6 estPtIFG_2781

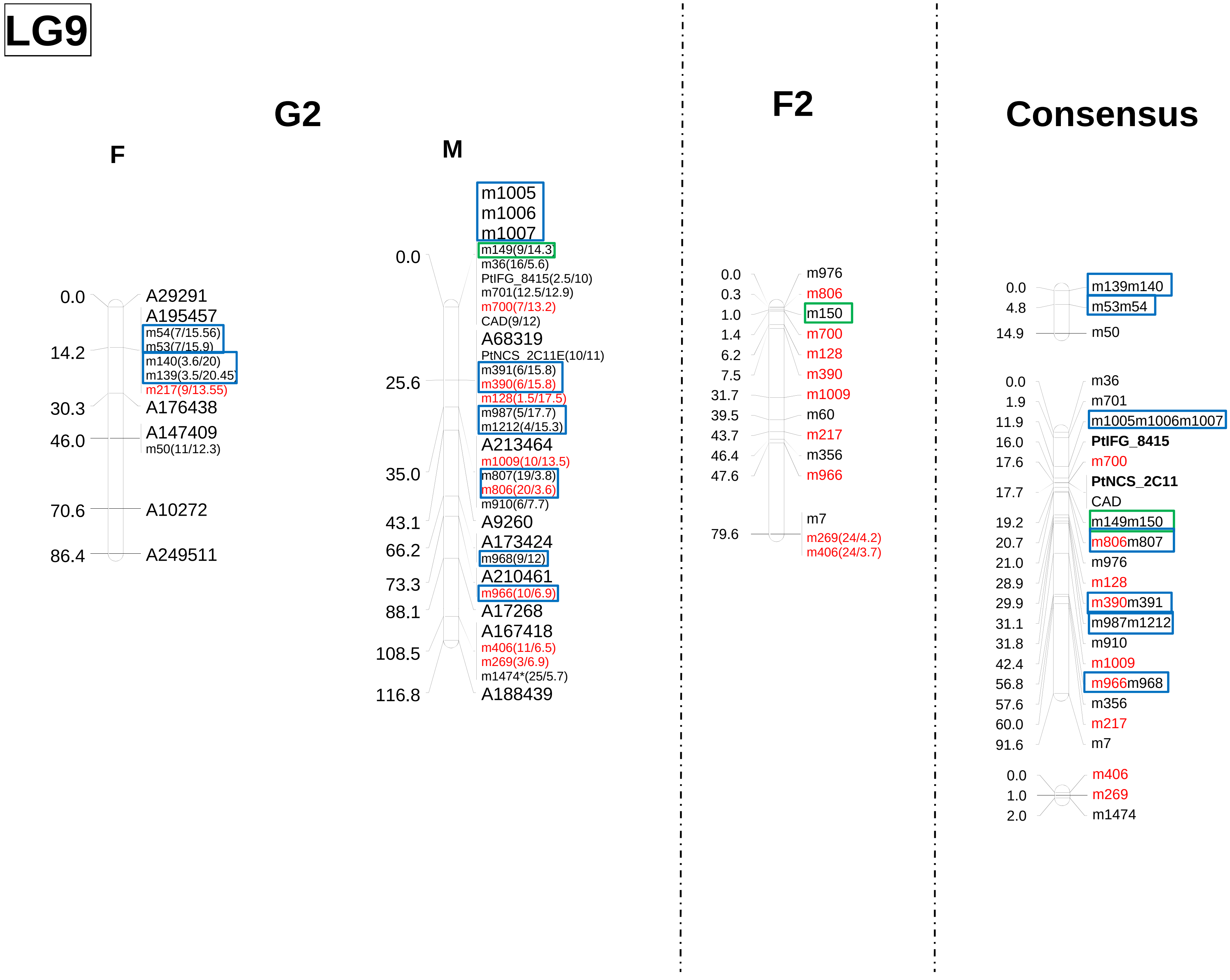
LG9
G2
F
0.0 A29291
A195457
m54(7/15.56)
m53(7/15.9)
14.2
m140(3.6/20)
m139(3.5/20.45)
m217(9/13.55)
30.3 A176438
46.0 A147409
m50(11/12.3)
70.6 A10272
86.4 A249511
M
m1005
m1006
m1007
0.0 m149(9/14.3)
m36(16/5.6)
PtIFG_8415(2.5/10)
m701(12.5/12.9)
m700(7/13.2)
CAD(9/12)
A68319
PtNCS_2C11E(10/11)
25.6 m391(6/15.8)
m390(6/15.8)
m128(1.5/17.5)
m987(5/17.7)
m1212(4/15.3)
A213464
m1009(10/13.5)
35.0 m807(19/3.8)
m806(20/3.6)
m910(6/7.7)
43.1 A9260
66.2 A173424
m968(9/12)
A210461
73.3 m966(10/6.9)
88.1 A17268
A167418
108.5 m406(11/6.5)
m269(3/6.9)
m1474*(25/5.7)
116.8 A188439
F2
0.0 m976
0.3 m806
1.0 m150
1.4 m700
6.2 m128
7.5 m390
31.7 m1009
39.5 m60
43.7 m217
46.4 m356
47.6 m966
m7
79.6 m269(24/4.2)
m406(24/3.7)
Consensus
0.0 m139m140
4.8 m53m54
14.9 m50
0.0 m36
1.9 m701
11.9 m1005m1006m1007
16.0 PtIFG_8415
17.6 m700
PtNCS_2C11
17.7 CAD
19.2 m149m150
20.7 m806m807
21.0 m976
28.9 m128
29.9 m390m391
31.1 m987m1212
31.8 m910
42.4 m1009
56.8 m966m968
57.6 m356
60.0 m217
91.6 m7
0.0 m406
1.0 m269
2.0 m1474

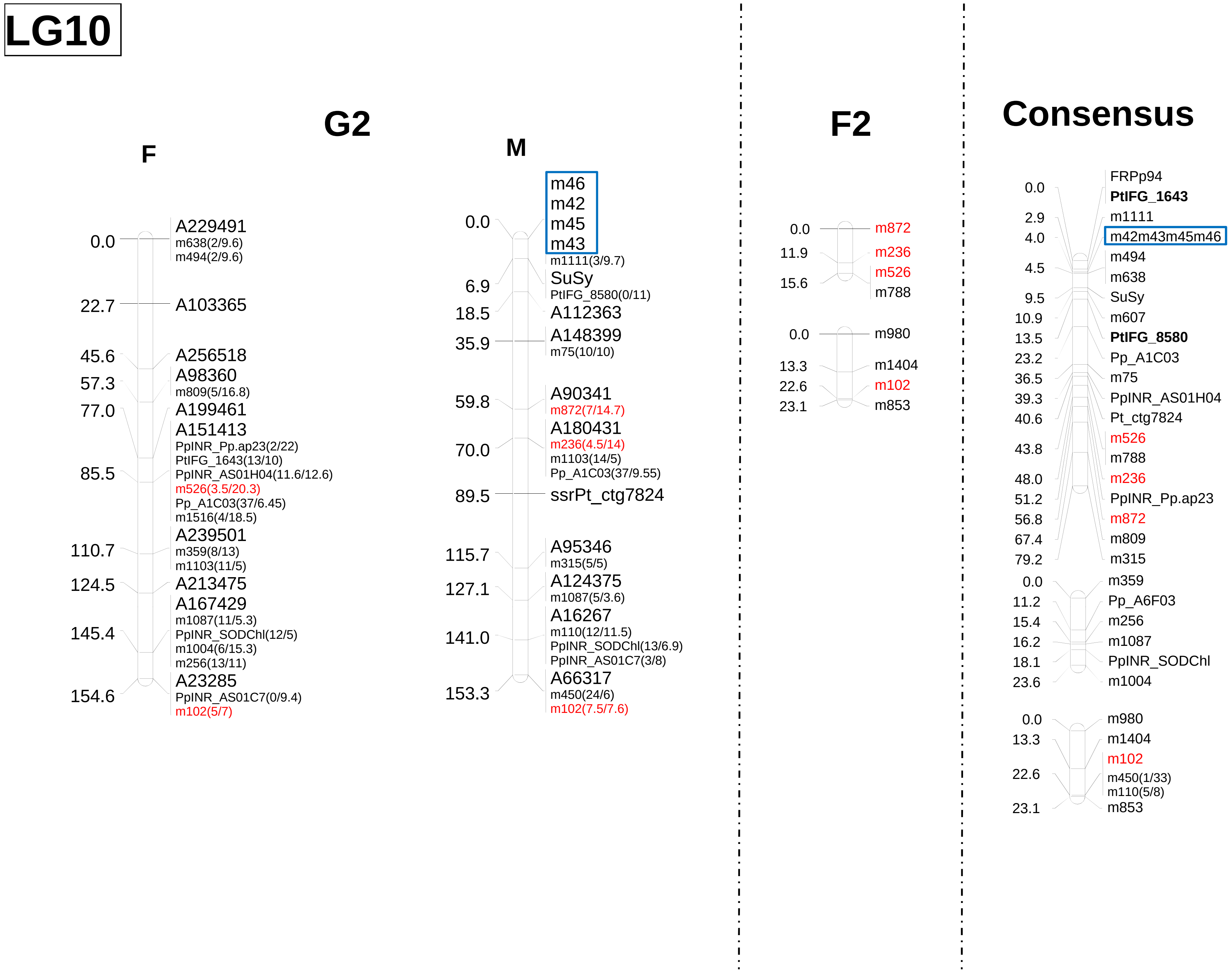
LG10
G2
F
M
F2
Consensus
0.0 A229491
m638(2/9.6)
m494(2/9.6)
22.7 A103365
45.6 A256518
57.3 A98360
m809(5/16.8)
77.0 A199461
A151413
PpINR_Pp.ap23(2/22)
PtIFG_1643(13/10)
85.5 PpINR_AS01H04(11.6/12.6)
m526(3.5/20.3)
Pp_A1C03(37/6.45)
m1516(4/18.5)
110.7 A239501
m359(8/13)
m1103(11/5)
124.5 A213475
A167429
m1087(11/5.3)
145.4 PpINR_SODChl(12/5)
m1004(6/15.3)
m256(13/11)
154.6 A23285
PpINR_AS01C7(0/9.4)
m102(5/7)
m46
m42
0.0 m45
m43
m1111(3/9.7)
6.9 SuSy
PtIFG_8580(0/11)
18.5 A112363
35.9 A148399
m75(10/10)
59.8 A90341
m872(7/14.7)
A180431
70.0 m236(4.5/14)
m1103(14/5)
Pp_A1C03(37/9.55)
89.5 ssrPt_ctg7824
115.7 A95346
m315(5/5)
127.1 A124375
m1087(5/3.6)
A16267
141.0 m110(12/11.5)
PpINR_SODChl(13/6.9)
PpINR_AS01C7(3/8)
153.3 A66317
m450(24/6)
m102(7.5/7.6)
0.0 m872
11.9 m236
15.6 m526
m788
0.0 m980
13.3 m1404
22.6 m102
23.1 m853
0.0 FRPp94
PtIFG_1643
2.9 m1111
4.0 m42m43m45m46
4.5 m494
m638
9.5 SuSy
10.9 m607
13.5 PtIFG_8580
23.2 Pp_A1C03
36.5 m75
39.3 PpINR_AS01H04
40.6 Pt_ctg7824
43.8 m526
m788
48.0 m236
51.2 PpINR_Pp.ap23
56.8 m872
67.4 m809
79.2 m315
0.0 m359
11.2 Pp_A6F03
15.4 m256
16.2 m1087
18.1 PpINR_SODChl
23.6 m1004
0.0 m980
13.3 m1404
m102
22.6 m450(1/33)
m110(5/8)
23.1 m853

LG11

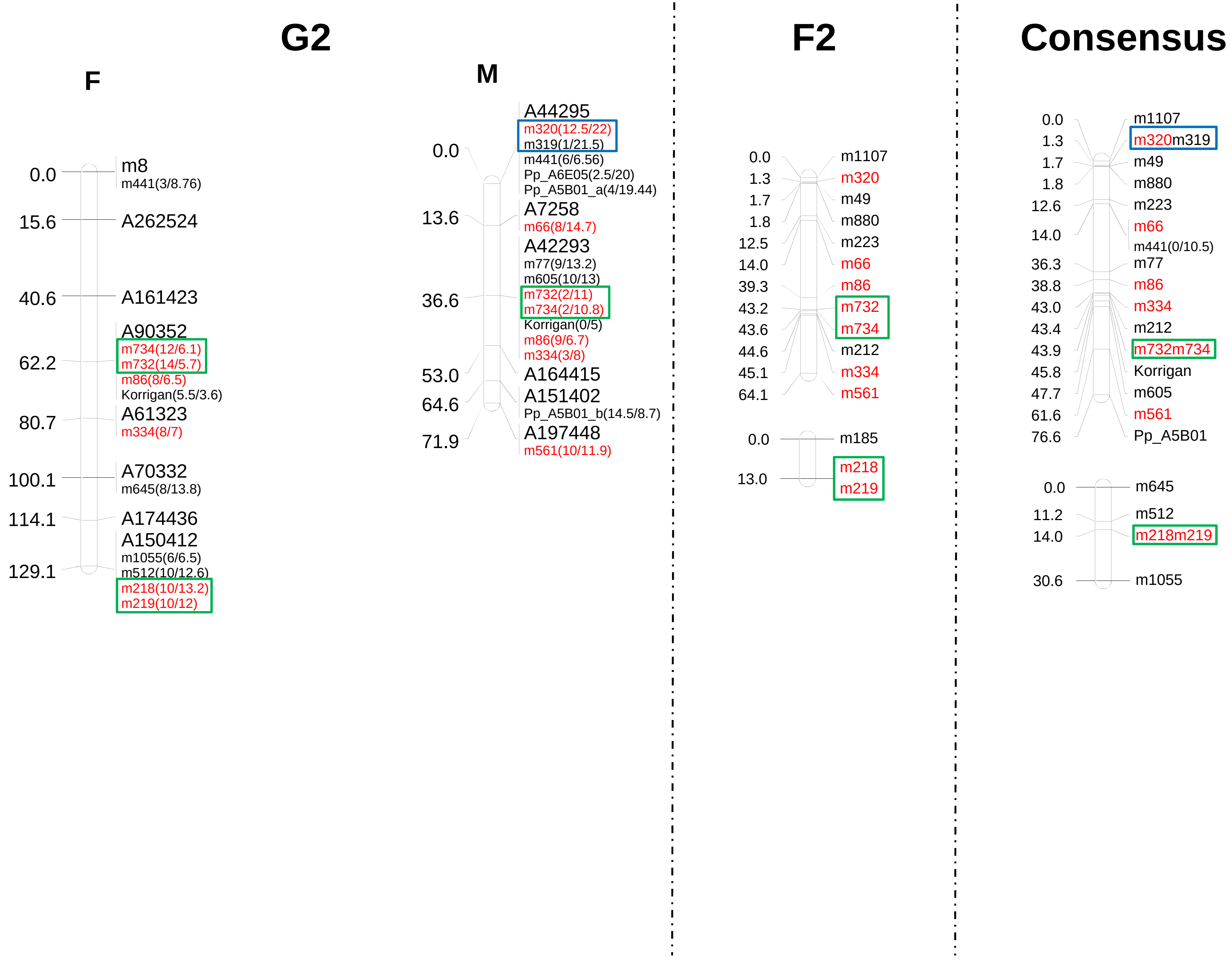
G2
F
0.0 m8 m441(3/8.76)
15.6 A262524
40.6 A161423
62.2 A90352 m734(12/6.1) m732(14/5.7) m86(8/6.5) Korrigan(5.5/3.6)
80.7 A61323 m334(8/7)
100.1 A70332 m645(8/13.8)
114.1 A174436
129.1 A150412 m1055(6/6.5) m512(10/12.6) m218(10/13.2) m219(10/12)
M
0.0 A44295 m320(12.5/22) m319(1/21.5) m441(6/6.56) Pp_A6E05(2.5/20) Pp_A5B01_a(4/19.44)
13.6 A7258 m66(8/14.7)
36.6 A42293 m77(9/13.2) m605(10/13) m732(2/11) m734(2/10.8) Korrigan(0/5) m86(9/6.7) m334(3/8)
53.0 A164415
64.6 A151402 Pp_A5B01_b(14.5/8.7)
71.9 A197448 m561(10/11.9)
F2
0.0 m1107
1.3 m320
1.7 m49
1.8 m880
12.5 m223
14.0 m66
39.3 m86
43.2 m732
43.6 m734
44.6 m212
45.1 m334
64.1 m561
0.0 m185
13.0 m218 m219
Consensus
0.0 m1107
1.3 m320m319
1.7 m49
1.8 m880
12.6 m223
14.0 m66 m441(0/10.5)
36.3 m77
38.8 m86
43.0 m334
43.4 m212
43.9 m732m734
45.8 Korrigan
47.7 m605
61.6 m561
76.6 Pp_A5B01
0.0 m645
11.2 m512
14.0 m218m219
30.6 m1055

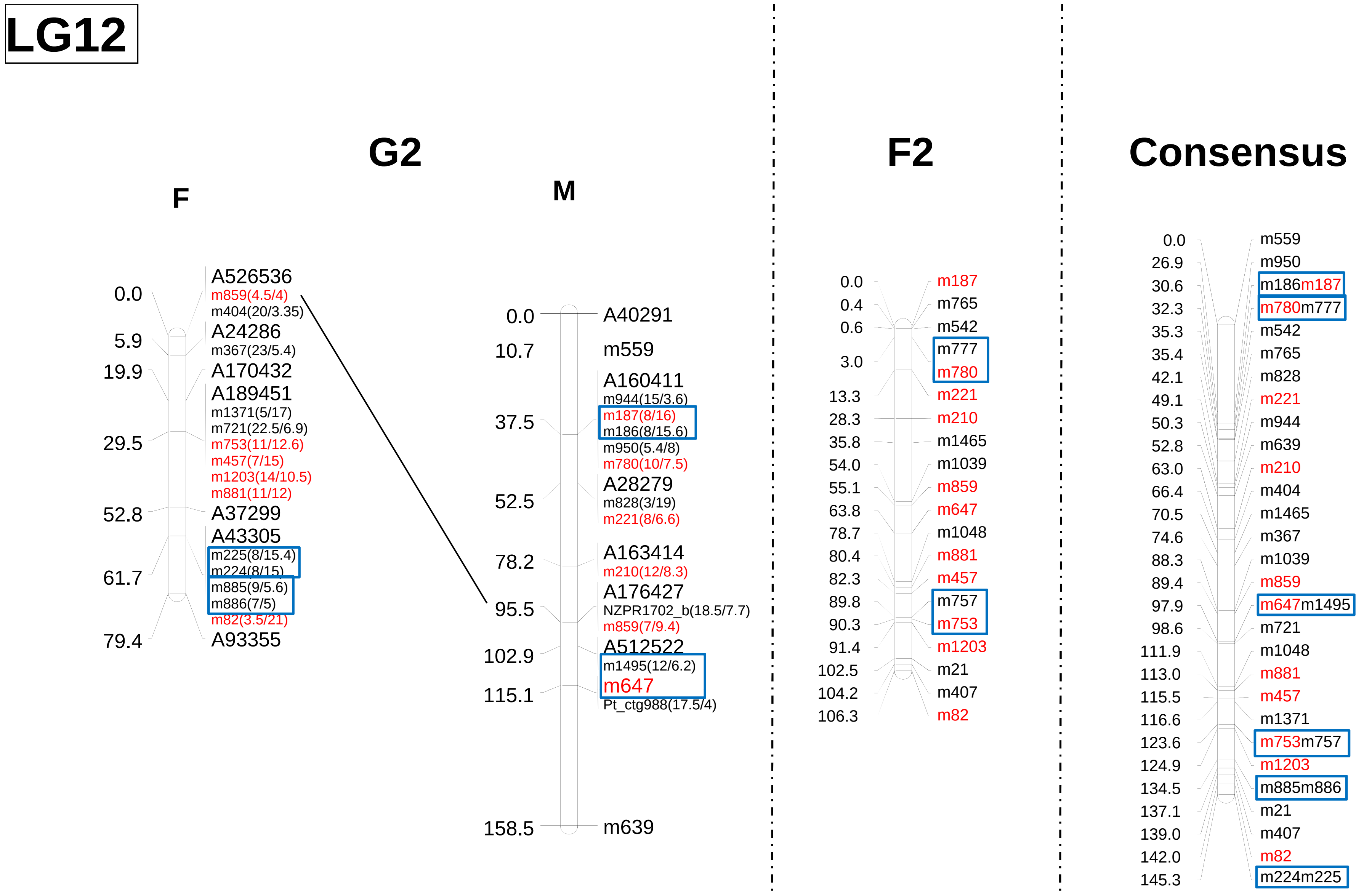
LG12
G2
F
M
F2
Consensus
0.0 A526536
m859(4.5/4)
m404(20/3.35)
5.9 A24286
m367(23/5.4)
19.9 A170432
A189451
m1371(5/17)
m721(22.5/6.9)
29.5 m753(11/12.6)
m457(7/15)
m1203(14/10.5)
m881(11/12)
52.8 A37299
A43305
m225(8/15.4)
m224(8/15)
61.7 m885(9/5.6)
m886(7/5)
m82(3.5/21)
79.4 A93355
0.0 A40291
10.7 m559
A160411
m944(15/3.6)
37.5 m187(8/16)
m186(8/15.6)
m950(5.4/8)
m780(10/7.5)
52.5 A28279
m828(3/19)
m221(8/6.6)
78.2 A163414
m210(12/8.3)
95.5 A176427
NZPR1702_b(18.5/7.7)
m859(7/9.4)
102.9 A512522
m1495(12/6.2)
115.1 m647
Pt_ctg988(17.5/4)
158.5 m639
0.0 m187
0.4 m765
0.6 m542
3.0 m777
m780
13.3 m221
28.3 m210
35.8 m1465
54.0 m1039
55.1 m859
63.8 m647
78.7 m1048
80.4 m881
82.3 m457
89.8 m757
90.3 m753
91.4 m1203
102.5 m21
104.2 m407
106.3 m82
0.0 m559
26.9 m950
30.6 m186m187
32.3 m780m777
35.3 m542
35.4 m765
42.1 m828
49.1 m221
50.3 m944
52.8 m639
63.0 m210
66.4 m404
70.5 m1465
74.6 m367
88.3 m1039
89.4 m859
97.9 m647m1495
98.6 m721
111.9 m1048
113.0 m881
115.5 m457
116.6 m1371
123.6 m753m757
124.9 m1203
134.5 m885m886
137.1 m21
139.0 m407
142.0 m82
145.3 m224m225
