# Supplementary figures and images for: Development and implementation of a highly-multiplexed SNP array for genetic mapping in maritime pine and comparative mapping with loblolly pine
Source: BMC Genomics. 2011 Jul 18;12:368. doi: 10.1186/1471-2164-12-368 (PMC3146957; doi:10.1186/1471-2164-12-368)

Additional file 6: Number of common SNP markers and contigs mapped in the G2 and F2 maps.

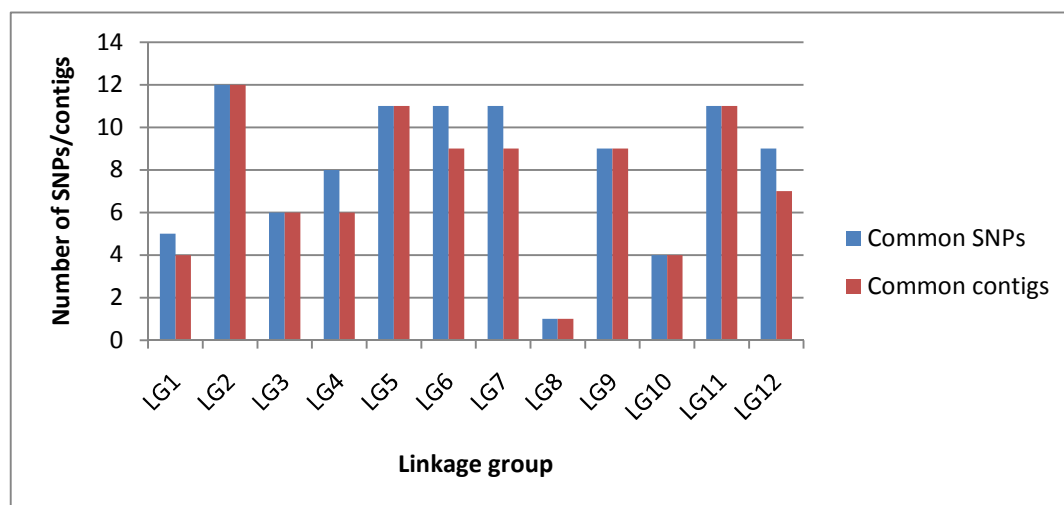

Supplement: Additional file 6 — Number of SNP markers and contigs mapped on the G2 and F2 linkage maps. [file 1471-2164-12-368-S6.PDF]
