# Supplementary material for: Development and implementation of a highly-multiplexed SNP array for genetic mapping in maritime pine and comparative mapping with loblolly pine
Source: BMC Genomics. 2011 Jul 18;12:368. doi: 10.1186/1471-2164-12-368 (PMC3146957; doi:10.1186/1471-2164-12-368)
Supplement: Additional file 7 — Number of orthologous markers mapped on the loblolly and maritime pine linkage groups. [file 1471-2164-12-368-S7.PDF]

Additional file 7: Number of orthologous markers mapped on the loblolly and maritime pine linkage groups.

Maritime pine	Loblolly pine *	Number of orthologous EST-P markers (Chagné *et al.* 2003)	Number of orthologous SNP markers (this study)	Total number of orthologous markers
LG1	LG1	1	-	1
LG2	LG2	3	10	13
LG3	LG3	4	1	5
LG4	LG4	4	1	6
LG5	LG5	4	2	6
LG6	LG6	7	6	13
LG7	LG7	1	3	4
LG8	LG8	2	1	3
LG9	LG9	2	5	7
LG10	LG10	2	8	10
LG11	LG11	-	4	4
LG12	LG12	-	5	5

* ID from Eckert et al. (2009)
